# Exploration of short-term predictions and long-term projections of Barents Sea cod biomass using statistical methods on data from dynamical models

**DOI:** 10.1371/journal.pone.0328762

**Published:** 2025-07-31

**Authors:** Mariko Koseki, Anne Britt Sandø, Geir Ottersen, Marius Årthun, Jan Erik Stiansen

**Affiliations:** 1 Geophysical Institute, University of Bergen, Bergen, Norway; 2 Bjerknes Centre for Climate Research, Bergen, Norway; 3 Institute of Marine Research, Bergen, Norway; 4 Centre for Ecological and Evolutionary Synthesis, Department of Biosciences, University of Oslo, Oslo, Norway; Woods Hole Oceanographic Institution, UNITED STATES OF AMERICA

## Abstract

This study aims to explore how well simple statistical modeling can generate short-term predictions and long-term projections of the total biomass of the Northeast Arctic stock of Atlantic cod (*Gadus Morhua*) inhabiting the Barents Sea. We examine the predictability of statistical models only based on hydrographic and lower trophic level biological variables from dynamical modeling. Simple and multiple linear regression models are developed based on gridded variables from the regional ocean model NEMO-NAA10km and the ecosystem model NORWECOM.E2E. This includes the essential environmental variables temperature, salinity, sea ice concentration, primary production and secondary production. The regression models are statistically evaluated to find variables that can capture variability in Barents Sea cod biomass. Finally, future total cod stock biomass is projected by applying the best found regression models to the range of downscaled IPCC climate scenarios from the coupled Intercomparison Project Phase 6 (CMIP6 Shared Socioeconomic Pathways; SSP1–2.6, SSP2–4.5, SSP5–8.5). Our prediction models are based on variables that affect cod both directly and indirectly. We find that several regression models have high prediction skill and capture the variations in total stock biomass of the Northeast Arctic cod well. Our results suggest that increased ocean temperature and abundant zooplankton may lead to a large cod stock. However, even if total stock biomass has a positive trend with an increase in copepods in the highest warming scenario SSP5–8.5, we found that it has a negative trend in the low emission scenario SSP1–2.6 when the regional ocean and ecosystem models show weak cooling and reduced zooplankton. We show that variability in essential environmental variables can provide a remarkably good first approximation to cod dynamics. However, to resolve the full picture other factors like fishing and natural mortality also need to be addressed explicitly.

## Introduction

The high spatiotemporal variability in many marine ecosystems is often linked to fluctuations in the physical environment (e.g., ocean temperature or currents). Variability is also often reflected in the abundance of a fish population and timing of critical events like spawning or feeding migration. This opens the door for developing and applying statistical models for predicting fish biomass. More than 100 years ago Helland-Hansen and Nansen (1909) [1] had a dream that one day we would be able to use climate information in predicting the development of fish populations. Since then, our understanding of how climate affects marine life has progressed substantially. However, making reliable “ecological forecast for marine resources” [2] is still very far from being straightforward. It is an important field to push forward as knowledge about the future status of fish stocks would give valuable information for a wide range of advisors and decision makers such as fisheries scientists, resource economists, policy experts, governmental managers and fishing industry.

Depending on the time scale of interest, we here use different expressions for future climate forecasts (with associated effects on marine ecosystems) based on commonly used expressions in climate research, namely climate prediction for short-term time scales and climate projection for long-term time scales. In both cases, we have used data from a regional dynamic ocean model [3] that is downscaled from a global climate model [4,5]. A dynamic ocean model is a numerical model that simulates the movement and development of ocean currents, temperature, salinity and other physical properties in the ocean by solving the fundamental equations of motion for fluids in a given grid for different layers in the ocean. The distance between these grid points constitutes the spatial resolution in the model, and the better the resolution, the more accurate the results. The same applies to the model’s time step.

Dynamic climate models can, by means of assimilation of observations and proper initialization, forecast the climate on different time scales that span time periods from seasons to decades [6], and predictions are often used to describe these forecasts. The climate can also be forecasted by using observation-based time series and multiple linear regression models [7]. The observation-based time series can either be used directly or they can be extracted from a dynamic ocean model simulation that is forced with realistic atmospheric forcing. Such short-term predictions for the Barents Sea are based on the fact that the water masses in the North Atlantic and the Nordic Seas move relatively slowly northwards towards the Barents Sea [8]. Anomaly high or low temperatures or salinity values in the North Atlantic therefore take several years to be transferred to the Barents Sea, and this provides a basis for predicting climate variability and associated effects on the marine ecosystem in the Barents Sea with a number of years [7]. Climate projections are based on the same type of global climate models, but they are driven by atmospheric forcing resulting from future emission scenarios related to different Shared Socioeconomic Pathways (SSPs; [9]). The focus is most often on multi-decadal time scales until the end of this century with particular interest in long-term variability and trends only.

Both climate and fishery influence fish stocks at different levels (e.g., [10]), with biological effects that can act on individuals, populations and in an ecosystem context. It should be recognized that there are accumulated biological, climatic and fishery impacts formed from complex direct and indirect pathways that also act on different time scales. There is also a lack of information forward in time. Quantifying uncertainty propagation when model complexity is high is also challenging. Therefore, the potential for simpler prediction methods should be investigated.

The Barents Sea is an open arcto-boreal shelf sea of around 1.4 million km^2^ located between 70 °N and 80 °N, off the Northeast Atlantic and north of Norway and north-western Russia. Interannual variability in ocean temperatures is strongly affected by the relatively warm Atlantic water masses flowing in from the southwest [11,12]. This year-to-year variability is largely determined by conditions during winter, the season when the differences in temperature, both between inflowing and local water masses and between atmospheric temperatures and sea surface temperature, are at their highest [11,13]. The Norwegian Atlantic Current (NwAC), which is the poleward extension of the North Atlantic Current (NAC), enters the Barents Sea through the Barents Sea Opening, located between the Bear Island and northern Norway [14]. The NAC/NwAC carries ocean heat from the subpolar North Atlantic towards the Arctic Ocean [8,15], and the inflow of Atlantic Water makes the Barents Sea partly ice-free ocean during winter [16,17]. Since the early 2000s, there has been a trend towards warming of the Barents Sea, related to changes in inflow of Atlantic water masses [12,18].

The Barents Sea is one of the biologically most productive seas in the world [19] with several large fish stocks, most prominently the world’s largest stock of Atlantic cod (*Gadus morhua*). The Barents Sea cod, also referred to as Northeast Arctic cod (NEA cod) is an important species in the Barents Sea both ecologically and commercially. NEA cod has its core habitat in the southern and central Barents Sea and its spawning area stretched along the northern Norwegian coast, with Lofoten as the main area. Spawning takes place during winter and the larvae drift with the currents into the Barents Sea, where they settle in the autumn, around September-October [20,21]. The cod spends the rest of their lifetime in the Barents Sea, except for the spawning migration of mature cod, back to the northern Norwegian coast.

However, during the lifecycle the cod changes its role in the ecosystem. As first-feeding larvae it depends on small zooplankton (mainly the first stages of the copepod *Calanus finmarchicus*; [22]). As it grows it feeds on larger and larger animals, first larger copepods and later fish and benthic organisms. The diet is quite wide, it eats basically what it can find [23]. This includes cannibalism. Capelin (*Mallotus villosus*), with its high fat content, seems to be a favorite prey, and in years with high capelin abundance the condition of cod is also found to be better [24]. Young NEA cod is an important prey for larger cod and several other fish species, seabirds and mammals. Older cod has few enemies, mainly larger cod and seals.

Environmental conditions, especially ocean temperature, have significant impact on many aspects of the cod population, including recruitment [25–27], distribution [28,29], and stock biomass [28,30]. The warm conditions in the region during ca 2004–2012 led to an unprecedented expansion of the cod’s habitat towards the northern and eastern Barents Sea [28,31] and high recruitment [27] and biomass [28].

The NEA cod fisheries have been important for over 1000 years. First only as coastal fishing at the spawning grounds in the spring, with the Lofoten fishery as the most famous. Since open ocean trawlers started all-year industrial fishing around the 1960s this has now become the major fishery. Fishing quotas are first set nationally between Russia and Norway, with a small share to a few other nations, before national quotas are divided to fleet groups and single vessels. Totally, fishing can take out up to as much as 30% of the fishable stock (i.e., above age 3 cod) yearly.

Årthun et al. (2017) [8] analyzed historical data and revealed a robust and statistically significant lagged relationship between poleward propagating ocean temperature anomalies along the northern extensions of the Gulf Stream and the Arctic climate variability. Building upon this connection and the strong co-variability between temperature anomalies and abundance of the Barents Sea cod stock, the predictions for important fish stocks are becoming feasible in our study region [7,32,33]. For example, statistically significant predictions of the cod total stock biomass seven years in advance were demonstrated by Årthun et al. (2018) [7]. Further, Koul et al. (2021) [32] developed a dynamical-statistical model system, using statistical models to link future NEA cod biomass to dynamical predictions of sea surface temperatures. The statistical predictions by Årthun et al. (2018) [7] and Koul et al. (2021) [32] are based only on ocean temperature. Changes in cod biomass are also related to, e.g., variable primary and secondary production [25,34,35].

Our aim is to explore how well just simple statistical modeling based on essential environmental variables time series from dynamic modeling can estimate total cod biomass. We then apply these to short-term predictions and long-term projections under the assumption that nothing else will change in the ecosystem (i.e., fishing, natural mortality etc.). By essential environmental variables we here mean physical variables such as temperature, salinity, sea ice and biological variables at lower trophic levels such as primary and secondary production.

Our approach is to utilize observational data and results from regional ocean and ecosystem hindcast model simulations to: 1) Construct statistical regression models for prediction of NEA cod biomass in the Barents Sea; 2) Apply the resulting statistical relationships in different downscaled scenarios to project future total cod stock biomass..

Input data to the statistical models includes gridded hydrographic values from the regional ocean model NEMO-NAA10km [3,36] and biological values from the ecosystem model NORWECOM.E2E [36–41]. The regression models are evaluated to find the variables that best capture variability in Barents Sea cod biomass. Finally, future total stock cod biomass in the Barents Sea is projected by using the best regression models with variables from a set of downscaled future climate scenarios (CMIP6 Shared Socioeconomic Pathways; SSP1–2.6, SSP2–4.5, SSP5–8.5). Future projections are typically provided by global atmosphere-ocean general circulation model NorESM2-MM [4,36]. However, such global models provide results with coarse resolution [40] and are often unreliable for projecting regional developments in fish populations [42]. Therefore, we build our cod biomass projections on time series from downscaled global models, using the NEMO-NAA10km and the NORWECOM.E2E models [36].

The Barents Sea and the marine ecosystem therein is exposed to many different processes related to the seasonal light variability, formation and melting of sea ice, wind-induced mixing, and exchange of heat and nutrients with neighboring ocean regions. Downscaled climate models in combination with low trophic ecosystem models provide projections of such climate exposures in addition to projections of primary and secondary production.

However, models can only generate results, which are already predetermined by the model equations [40]. It is important to see if the models can capture the impacts on ecosystem structure which may be caused by climate change in the future projections.

In this paper we show several regression models that have high prediction skill and can capture the variations in total stock biomass of the Northeast Arctic cod well. Our results suggest that increased ocean temperature and abundant zooplankton may lead to a large cod stock. However, in the low emission scenario (SSP1–2.6) when the regional ocean and ecosystem models show weak cooling and reduced zooplankton we found a negative trend for total stock biomass of cod. In general, despite a simple approach to a complex issue, our results shows that basic environmental variables can provide a remarkably good first approximation to cod dynamics. However, it is not and should not be good enough to fully resolve the picture.

## Materials and methods

### Locations of focus areas

**Fig 1:**
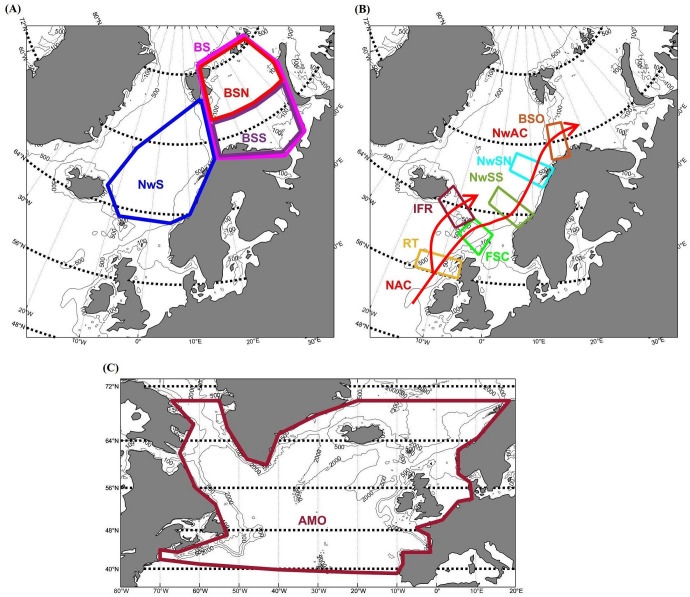
Locations of focus areas. Boxes and polygons indicate the areas where variables are extracted. Abbreviations are defined below: **(A)** BS: Barents Sea, BSN: Barents Sea-North, BSS: Barents Sea-South, NwS: Norwegian Sea, **(B)** Focus areas along the North Atlantic Current (NAC) and the Norwegian Atlantic Current (NwAC); BSO: Barents Sea Opening, NwSN: Norwegian Sea-North, NwSS: Norwegian Sea-South, FSC: Faroe-Shetland Channel, IFR: Iceland-Faroe Ridge, RT: Rockall Trough, **(C)** AMO index: Atlantic Multidecadal Oscillation index.

Water masses anomalies in temperature and salinity are advected with the main current systems. Such anomaly advection can take years from the North Atlantic to the Barents Sea (e.g., [43]). Locations of the focus areas (see Fig 1) are therefore chosen as larger sea areas or as polygons along the transport route advecting into and intersecting the distribution area of NEA cod. The largest chosen areas are the whole Barents Sea (BS) and the Norwegian Sea (NwS). We also divide the Barents Sea into two parts, the northern part: Barents Sea-North (BSN) and the southern part: Barents Sea-South (BSS) (Fig 1A). Further, several polygons along the NAC and the NwAC are also chosen for our analyses (Fig 1B): Barents Sea Opening (BSO), Norwegian Sea-North (NwSN), Norwegian Sea-South (NwSS), Faroe-Shetland Channel (FSC), Iceland-Faroe Ridge (IFR), and Rockall Trough (RT).

### Total stock biomass of the NEA cod in the barents sea

**Fig 2:**
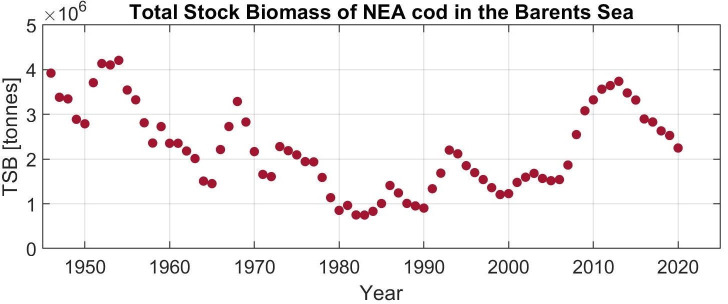
Total stock biomass of the NEA cod in the Barents Sea (TSB) 1946-2020.

Total stock biomass of the NEA cod in the Barents Sea (TSB) from 1946 to 2020, obtained from the ICES Arctic Fisheries Working Group annual report [44], is shown in Fig 2. For correlations and regressions, we have used the cod biomass anomaly in the calculation.

### Hydrographic data and AMO

**Fig 3:**
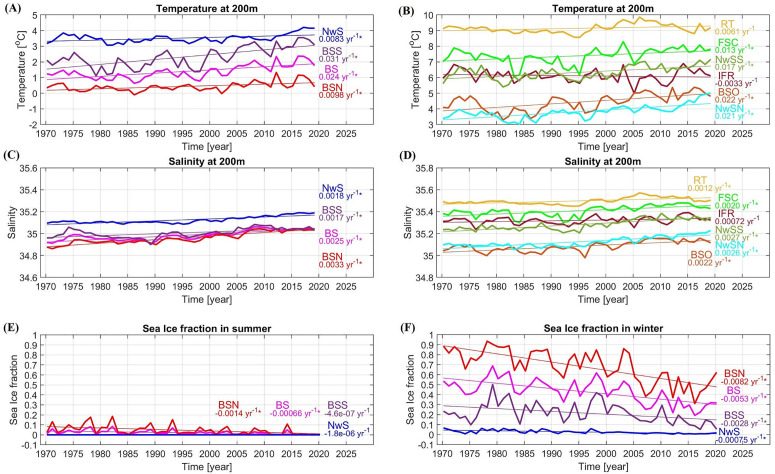
Trends of hydrographic time series in each location. Trends of **(A)** temperature in the Barents Sea and the Norwegian Sea, **(B)** temperature along the NAC/NwAC, **(C)** salinity in the Barents Sea and the Norwegian Sea, **(D)** salinity along the NAC/NwAC, **(E)** sea ice fraction in summer (September), and **(F)** sea ice concentration in winter (March). Linear trends are calculated using the least squares methods, and significance of trend is calculated with the Mann-Kendall test. Significant trends (*p* < 0.05) are indicated by an asterisk. Abbreviations of location names are defined in Fig 1.

Gridded data of temperature, salinity, and sea ice concentration are taken from the hindcast simulation of a regional ocean model: NEMO-NAA10km (NAA stands for North Atlantic & Arctic) between 1970 and 2019. More details on the set-up for the hindcast simulation of NEMO-NAA10km are provided in [3]. Time series of winter temperature and salinity at 200 m depth, and sea ice concentration in summer (September) and winter (March) are made by calculating the average of each variable in each focus area, defined in Fig 1. Time series and trends of hydrography and sea ice concentration are shown in Fig 3. Moreover, S1 - S3 Figs illustrate the patterns of hydrographic data in the North Atlantic and the Arctic.

The Atlantic Multidecadal Oscillation (AMO; [45]) index is in this paper defined as the unsmoothed winter sea surface temperature from 42°N in the western and 39°N in the eastern North Atlantic to 70˚N (Fig 1C), obtained from a hindcast simulation of a regional ocean model: NEMO-NAA10km. The calculations are according to Levitus et al. (2009) [46]. The AMO index is also used as a predictor because hydrography in the Barents Sea reflects large-scale changes in the North Atlantic, as captured by the AMO index [46], and the AMO influences marine species (e.g., phytoplankton, zooplankton, fish) through both direct and indirect effects [47].

### Biological data

Biological gridded data of gross primary production (diatom and flagellate) and gross secondary production (micro and meso zooplankton) is obtained from an ecosystem model: NORWECOM.E2E [36] between 1970 and 2019. The hindcast simulation with NORWECOM.E2E is run with the physical ocean fields (velocities, salinity, temperature, sea surface height, and sea-ice) from the NEMO-NAA10km together with observation based atmospheric forcing [3].

**Fig 4:**
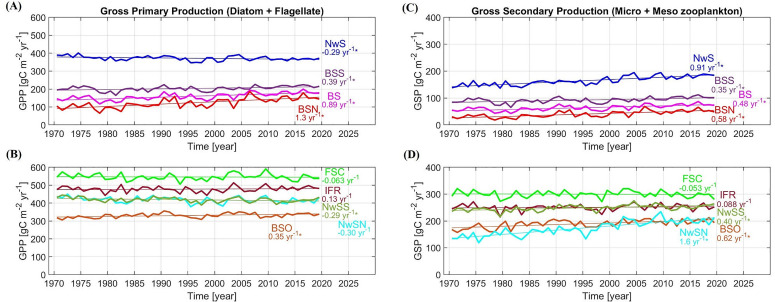
Trends of biological time series in each location. Trends of **(A)**, **(B)** gross primary production (GPP), and **(C)**, **(D)** gross secondary production (GSP), are shown. Linear trends are calculated using the least squares methods, and significance of trend is calculated with the Mann-Kendall test. Significant trends (*p* < 0.05) are indicated by an asterisk. Abbreviations of location names are defined in Fig 1. Note that time series of GPP and GSP at RT are not available because this focus area is located outside of the ecosystem model domain.

Time series of annual gross primary production (GPP) and gross secondary production (GSP) are made by averaging each variable in each focus area, defined in Fig 1. Trends of biological time series are shown in Fig 4. Moreover, S4 and S5 Figs illustrate the patterns of biological data in the North Atlantic and the Arctic. Note that time series of GPP and GSP at Rockall Trough (RT) are not available because this location is outside of the ecosystem model domain.

### Lag correlation analysis

Correlation analysis is performed between TSB and hydrographic/biological time series, with time lag range between 0 and 10 years for all variables and time lags. The time lag with maximum correlation is chosen if there is a correlation between TSB and variables. If there are more than one peak of correlation, the time lag with the first peak lagged correlation is chosen. The anomalies of TSB, temperature, salinity, GPP, GSP are used for lag correlation analysis and are relative to 1970–2019. Sea ice concentration, ranging from 0 to 1, is also used for lag correlation analysis for the same period.

Several locations along the Norwegian Atlantic Current are chosen for correlation analysis because the prediction potential of anomalies advected with the Atlantic Water can be exploited to study corresponding impacts of upstream anomalies on cod biomass in the Barents Sea. The importance of propagating hydrographic anomalies for fish stock was reported [43]. Moreover, prediction models were constructed based on the northward propagation of hydrography along the North Atlantic Current towards the Barents Sea [7]. Therefore, we follow the method in Årthun et al. (2018) [7] in this study.

In addition, we are looking at stock biomass. Average age of NEA cod is around 6 years, and it can often reach over 13 years age (13 years and older is the oldest year class used in the stock assessment; [44]). Spawning success can be linked to maternal conditions, which again can be set the previous year. On top of that, climate signals can use 2–4 years to propagate from the North Atlantic to the Barents Sea [7]. We therefore feel that using a max time span of 10 years is within the range of processes affecting one lifecycle.

### Linear regression models

To predict TSB, simple and multiple linear regression models are constructed with different variables obtained from the regional ocean- and ecosystem models. The general equation of the linear regression model with time lag is given as


$$y_{a}=\alpha_{0}+\alpha_{1}x_{1,a-b}+\alpha_{2}x_{2,a-b}+\ldots +\alpha_{k}x_{k,a-b}+\varepsilon$$

where *y*_*a*_ is the response variable/predictand (TSB; year = *a*), *α*_0_ is the *y* intercept, *α*_*k*_ is the regression coefficient, *x*_*k*, *a*-*b*_ is the explanatory variable/predictor (e.g., temperature, salinity, GPP etc.), *b* is the time lag in year(s), *k* is the number of explanatory variables, and *ε* is the residual [7,48]. The time lag (*b* year(s)) is based on the lag correlation analysis.

In this study multiple regression models (with and without interaction term) are limited to two predictors (details on regression analysis are provided in S1 File) to avoid overfitting and keep the analyses simple.

### Evaluation of linear regression models

After the regression models are constructed, these models are evaluated to find which variables to be used to predict TSB well, namely, to determine the best regression model that can capture variations in TSB.

To evaluate regression models, the statistical values, such as coefficient of determination (*R*^2^; ranges from 0 to 1), *F*-statistics, *p*-value, residual sum of squares (*RSS*) are calculated (details on the calculations are provided in S1 File).

The *F*-statistics and the *p*-value can be used to assess the significance of the regression models [48,49].

Further, Akaike Information Criterion (AIC) and delta AIC [50] are calculated to compare different possible models and select the best model to fit the response variable. In this study, this criterion is used in model selection to minimize the number of “independent” explanatory variables, as well as to prevent selecting overfitting models. In other words, the AIC is used to determine the number of explanatory variables, not used to compare the value of AIC for each model. Therefore, the models with high *R*^2^ are basically considered to be the best-fit models even though the delta AIC is high. Furthermore, the models which are based on biological variables with high *R*^2^ are considered to be the best-fit models even though the models have higher delta AIC because models without biological support should not be included in the set of candidate models [50].

Moreover, to evaluate the regression coefficients of the models, statistics of the regression coefficient for each regression model are also calculated (details on the calculations are provided in S1 File).

Multicollinearity is measured using variance inflation factor (*VIF*; only for multiple regression models; [48]) when the best model is selected. Multicollinearity is a phenomenon where one explanatory variable is highly correlated with one or more of the other explanatory variables in a multiple regression model, and it leads to undesirable consequences (e.g., coefficients may have unrealistic opposite sign, coefficients of slope are not stable; [48]). To detect multicollinearity in the multiple regression models, the *VIF* for each variable is calculated. The *VIF* for the *j*^th^ explanatory variable is given as


$${VIF}_{j}=\frac{1}{1-{R_{j}}^{2}}$$

where *R*_j_^2^ is the *R*^2^ on all the other explanatory variables. The minimum value of the *VIF* is 1, and *VIF* = 1 means that there is no correlation between explanatory variables in the model. In general, when the *VIF* exceeds 5 or 10, high multicollinearity is observed between this variable and the others. Serious problems occur when the value of *VIF* is greater than 10 [48].

### Application of regression models to downscaled climate projections

To project TSB in the future, variables used in the regression models are taken from downscaled models for three different IPCC climate scenarios: low (SSP1–2.6), medium (SSP2–4.5), and high (SSP5–8.5) emission [9]. According to Burgess et al. (2023) [51], the high-emission scenario SSP5–8.5 seems to be implausible with too high global warming. On the other hand, Chylek et al. (2024) [52] compared NorESM2 to the average of seven other CMIP6 models and found that NorESM2 underestimates the future Arctic warming. With this in mind, we think that using the SSP5–8.5 from NorESM2 can still be representative for a pessimistic high-emission scenario in the Barents Sea region.

**Fig 5:**
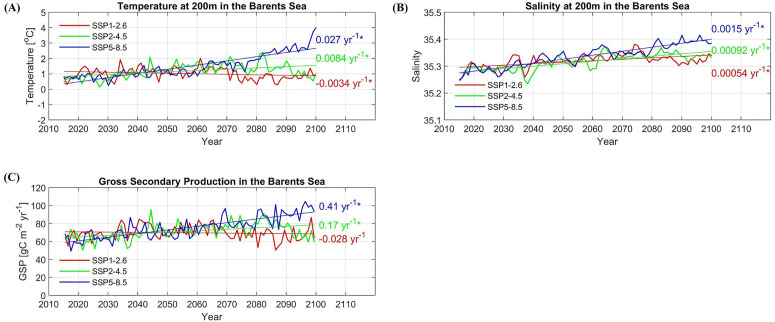
Trends of hydrographic/biological time series in future climate scenarios: SSP1-2.6, SSP2-4.5, SSP5-8.5. Trends of **(A)** temperature at 200 m depth, **(B)** salinity at 200m depth, and **(C)** gross secondary production (GSP) in the Barents Sea between 2015 and 2100, are shown. Linear trends are calculated using the least squares methods, and significance of trend is calculated with the Mann-Kendall test. Significant trends (*p* < 0.05) are indicated by an asterisk.

Future hydrographic (temperature and salinity at 200 m) and biological (GPP and GSP) gridded data between 2015 and 2100 are obtained from NEMO-NAA10km [3] and NORWECOM.E2E [40,41], respectively. In this study, the climate scenarios from the NorESM2 (the second version of the Norwegian Earth System Model; [5,53]) are downscaled using the regional ocean model: NEMO-NAA10km to produce the physical forcing for the ecosystem model: NORWECOM.E2E. More details on the model set-up for downscaling are provided in [41]. Trends of future hydrographic/biological time series are shown in Fig 5.

### Method summary

The intention of our approach was never to find the optimal model for Total NEA cod biomass, but to see how well a simple statistical bottom-up approach would do. Therefore, we choose few variables in the regression models (no more than 2) and even though we ran quite a few correlations and regressions for good fit we also looked at plausibility (i.e., our expert “gut” feeling) in addition to the set of criteria in the final pick.

Our approach can shortly be listed as:

- Focus areas are selected along the pathway of water mass advection and stock distribution areas.
- Historic time series are picked based on potential for long projections: Climate essential environmental variables (temperature, salinity, sea ice and AMO) time series are calculated from the numerical model NEMO-NAA10km (physics and AMO). Biological essential environmental time series (GPP and GSP) are taken from the ecosystem model NORWECOM.E2E (GPP and GSP). Cod time series are taken from ICES assessment.
- Alle the time series are cross correlated with time lag 0–10 years, and the time lag with the best correlations (correlation coefficient > 0.65) is chosen.
- Based on the lag correlation analysis, simple/multiple linear regression models for TSB are constructed by using hydrographic/biological variables with a time lag that has a high correlation (presented in Table 1 and in S1 Table).
- The regression models are evaluated to find the variables that best capture variability in Barents Sea cod biomass. Models with high *R*^2^ is basically considered to be the best-fit models, but other criteria such as *p*-value and *VIF* are also considered. For multiple regressions, also AIC is used, and *VIF* is used to detect multicollinearity. Test with *p*-value and *F*-statistics to assess the significance of the regression models.
- Future total stock cod biomass in the Barents Sea is projected by using the best regression models with variables from a set of downscaled (from global climate models by NEMO-NAA10km and NORWECOME.E2E) future climate scenarios SSP1–2.6, SSP2–4.5, SSP5–8.5).

**Table 1:** Lag correlation analysis between TSB and hydrographic/biological time series in the Barents Sea/Norwegian Sea, along the NAC/NwAC, AMO index.

Focus area	Temperature at 200m depth			Focus area	Salinity at 200m depth		
	*r*	*p*-value	Time lag [year(s)]		*r*	*p*-value	Time lag [year(s)]
BS	0.80	*p* < 0.001	3	BS	0.81	*p* < 0.001	1
BSN	0.62	*p* < 0.001	4	BSN	0.73	*p* < 0.001	1
BSS	0.77	*p* < 0.001	3	BSS	0.79	*p* < 0.001	1
NwS	0.50	*p* < 0.001	0	NwS	0.78	*p* < 0.001	5
FSC	0.50	*p* < 0.001	0	FSC	0.62	*p* < 0.001	6
BSO	0.76	*p* < 0.001	3	BSO	0.82	*p* < 0.001	3
NwSN	0.61	*p* < 0.001	1	NwSN	0.75	*p* < 0.001	7
NwSS	0.64	*p* < 0.001	4	NwSS	0.80	*p* < 0.001	6
RT	0.74	*p* < 0.001	6	RT	0.80	*p* < 0.001	6
**Focus area**	**Sea ice concentration in winter**						
	***r***	***p*-value**	**Time lag [year(s)]**				
BS	−0.75	*p* < 0.001	2				
BSN	−0.73	*p* < 0.001	2				
BSS	−0.59	*p* < 0.001	1				
NwS	−0.59	*p* < 0.001	2				
**Focus area**	**Gross primary production (GPP)**			**Focus area**	**Gross secondary production (GSP)**		
	***r***	***p*-value**	**Time lag [year(s)]**		***r***	***p*-value**	**Time lag [year(s)]**
BS	0.67	*p* < 0.001	2	BS	0.67	*p* < 0.001	2
BSN	0.63	*p* < 0.001	3	BSN	0.65	*p* < 0.001	3
BSS	0.60	*p* < 0.001	2	BSS	0.62	*p* < 0.001	2
–	–	–	–	NwS	0.73	*p* < 0.001	6
BSO	0.46	2.00E-03	8	BSO	0.52	*p* < 0.001	8
–	–	–	–	NwSN	0.74	*p* < 0.001	7
–	–	–	–	NwSS	0.56	*p* < 0.001	9
**AMO index**							
***r***	***p*-value**	**Time lag [year(s)]**					
0.71	*p* < 0.001	6					

Maximum correlation coefficient (*r*) between TSB and variable, *p*-value and time lag which has maximum correlation are given. Note that time series of GPP and GSP at Rockall Trough (RT) are not available because this focus area is located outside of the ecosystem model domain. For simplicity, only significant correlations (*p* < 0.01) are shown. All results from lag correlation analysis are provided in S1 Table.

## Results

### Lag correlation analysis

**Fig 6:**
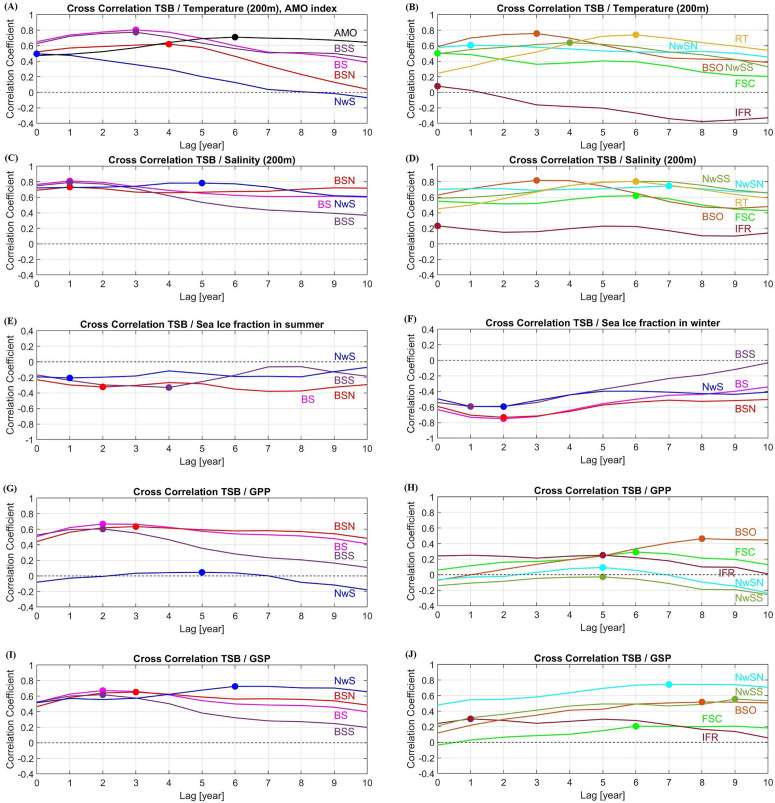
Lag correlation analysis between total stock biomass (TSB) and hydrographic/biological variables. Cross correlations between TSB and **(A)** temperature at 200m depth in the Barents Sea (BS) and the Norwegian Sea (NwS), and AMO index, **(B)** temperature along the NAC/NwAC, **(C)** salinity at 200m depth at BS and NwS, **(D)** salinity along the NAC/NwAC, **(E)** sea ice fraction in summer at BS and NwS, **(F)** sea ice fraction in winter at BS and NwS, **(G)** GPP at BS and NwS, **(H)** GPP along the NAC/NwAC, **(I)** GSP at BS and NwS, and **(J)** GSP along the NAC/NwAC, are shown. The spots show the maximum correlation. Abbreviations of focus area names are defined in Fig 1.

Several locations have a high and significant correlation between TSB and hydrographic/biological variables (Table 1, S1 Table, Fig 6).

This applies in particular to TSB and temperature time series in the Barents Sea with a lag of 3 years (*r* = 0.80; Table 1, Fig 6A), and to TSB and salinity time series in the Barents Sea with a lag of 1 year (*r* = 0.81; Table 1, Fig 6C). The correlation between the AMO index and TSB is also high with a lag of 6 years (*r* = 0.71; Table 1, Fig 6A). There is a negative correlation between TSB and sea ice concentration in winter in the Barents Sea with time lag of 2 years (*r* = −0.75; Table 1, Fig 6F) while correlation between TSB and sea ice concentration in summer is low in both the Barents Sea and the Norwegian Sea as expected (S1 Table, Fig 6E). The correlation between TSB and GPP/GSP is also significant in several locations (Table 1, Fig 6G–J).

### Regression analysis

Based on the lag correlation analysis, simple/multiple linear regression models for TSB are constructed by using hydrographic/biological variables with a time lag that has a high correlation (Table 2, S2 and S3 Tables).

**Table 2:** List of simple/multiple regression models for total stock biomass of the NEA cod in the Barents Sea (TSB), statistics of the regression models and equations of best-fit models to TSB.

Simple regression models																
Model No.		Variable(s)				Focus area			*R* ^2 a^	*p*-value			AIC ^b^		delta AIC ^c^	
		*x*_1_			Time lag											
1-1-1		Temperature (200 m)			3	BS			0.65	*p* < 0.001			1374.63		13.53	
1-2-1		Salinity (200 m)			1	BS			0.66	*p* < 0.001			1429.55		68.45	
1-3-1		Sea Ice concentration (summer)			2	BS			0.10	2.51E-02			1447.53		86.43	
1-4-1		Sea Ice concentration (winter)			2	BS			0.56	*p* < 0.001			1413.10		52.00	
1-5-1		GPP			2	BS			0.45	*p* < 0.001			1424.56		63.46	
1-6-1		GSP			2	BS			0.45	*p* < 0.001			1424.06		62.96	
**Multiple regression models**																
Model No.	Variable			Variable				interaction term			*R* ^2^	*p*-value		AIC		delta AIC
	*x*_1_		Time lag	*x*_2_			Time lag									
2-1	Salinity (200 m)		1	Temperature (200m)			3	With			0.75	*p* < 0.001		1362.94		1.85
**2-2**	Salinity (200 m)		1	Temperature (200m)			3	Without			0.75	*p* < 0.001		1361.10		0.00
2-3	Salinity (200 m)		1	Sea Ice concentration (summer)			2	With			0.68	*p* < 0.001		1401.87		40.77
2-4	Salinity (200 m)		1	Sea Ice concentration (summer)			2	Without			0.68	*p* < 0.001		1400.05		38.95
2-5	Salinity (200 m)		1	Sea Ice concentration (winter)			2	With			0.72	*p* < 0.001		1396.17		35.07
**2-6**	Salinity (200 m)		1	Sea Ice concentration (winter)			2	Without			0.72	*p* < 0.001		1394.23		33.13
2-7	Temperature (200m)		3	GPP			2	With			0.69	*p* < 0.001		1372.74		11.64
**2-8**	Temperature (200m)		3	GPP			2	Without			0.68	*p* < 0.001		1371.57		10.48
2-9	Temperature (200m)		3	GSP			2	With			0.69	*p* < 0.001		1372.14		11.04
**2-10**	Temperature (200m)		3	GSP			2	Without			0.69	*p* < 0.001		1370.62		9.53
**Statistical best-fit models to TSB**																
**Model No.**	**Equation**															
**2-2**	${TSB}_{a}=1.1\times 10^{7}\times {Salinity}_{a-1}+7.9\times 10^{5}\times {Temperature}_{a-3}+2.1\times 10^{4}$															
**2-6**	${TSB}_{a}=1.2\times 10^{7}\times {Salinity}_{a-1}-2.3\times 10^{6}\times {Sea\;ice\;concentration}_{a-2}+9.9\times 10^{5}$															
**2-8**	${TSB}_{a}=1.4\times 10^{6}\times {Temperature}_{a-3}+1.1\times 10^{4}\times {GPP}_{a-2}+7.3\times 10^{4}$															
**2-10**	${TSB}_{a}=1.4\times 10^{6}\times {Temperature}_{a-3}+2.2\times 10^{4}\times {GSP}_{a-2}+7.2\times 10^{4}$															

For simplicity, only simple regression models, based on one variable taken in the Barents Sea and multiple regression models, based on a combination of two variables obtained in the Barents Sea, are listed here. Bold shows the models that we find statistically best-fit to TSB. More details and other statistics are provided in S2 and S3 Tables.

^a^ Coefficient of determination

^b^ Akaike’s Information Criterion

^c^ The difference from minimum value of AIC

Several regression models are significant at 0.1% significance level (Table 2, see also S2 and S3 Tables), and predict variation in TSB several years in advance. For instance, the model No. 1-1-1, based on temperature time series in the Barents Sea, can explain 65% of variation in TSB 3 years in advance (Table 2, S6 Fig A), and the model No. 1-4-1 (based on winter sea ice concentration in the Barents Sea) can explain 56% of TSB variation 2 years in advance (Table 2, S6 Fig F). Several simple linear regression models, based on GPP or GSP, can also capture variability in TSB well, with a significant level better than 0.01 (Table 2, S2 Table, S6 Fig G – J).

Multiple regression models that include temperature as a predictor (No. 2−1, 2−2, 2−7, 2−8, 2−9, 2−10) can explain up to 75% of variation in TSB (Table 2, S7 Fig). Moreover, the multiple regression models can predict variation in TSB 1−2 years in advance. For instance, the model No. 2−1 (based on temperature/salinity) can predict variation in TSB 1 year in advance because salinity in the Barents Sea leads TSB by 1 year and temperature in the Barents Sea leads TSB by 3 years (Table 2).

### Evaluation of linear regression models

**Fig 7:**
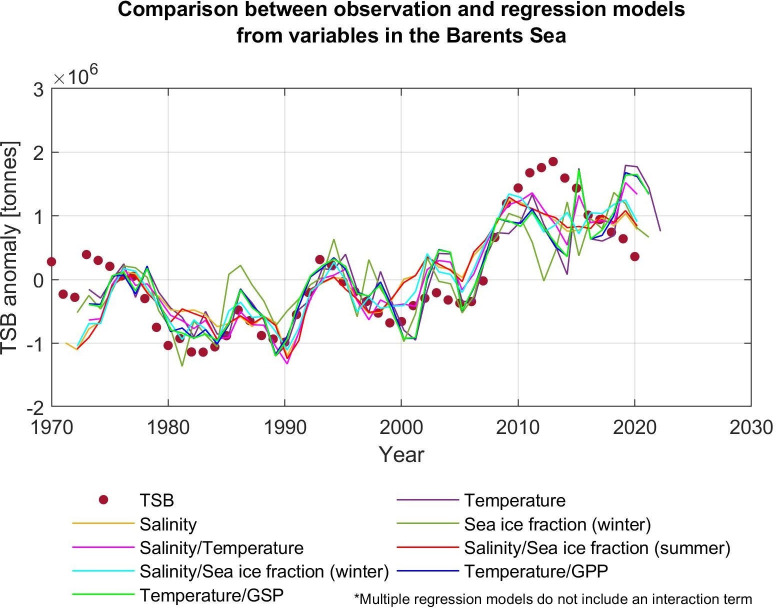
Comparison between observations and simple/multiple regression models. Spots show the total stock biomass of the NEA cod (TSB) from ICES assessment, and solid lines show TSB estimated by simple/multiple regression models (0.5 > *R*^2^). Each regression model is constructed by one or two variables. For simplicity, only multiple regression models without an interaction term are plotted. Anomalies are relative to 1970-2019.

**Fig 8:**
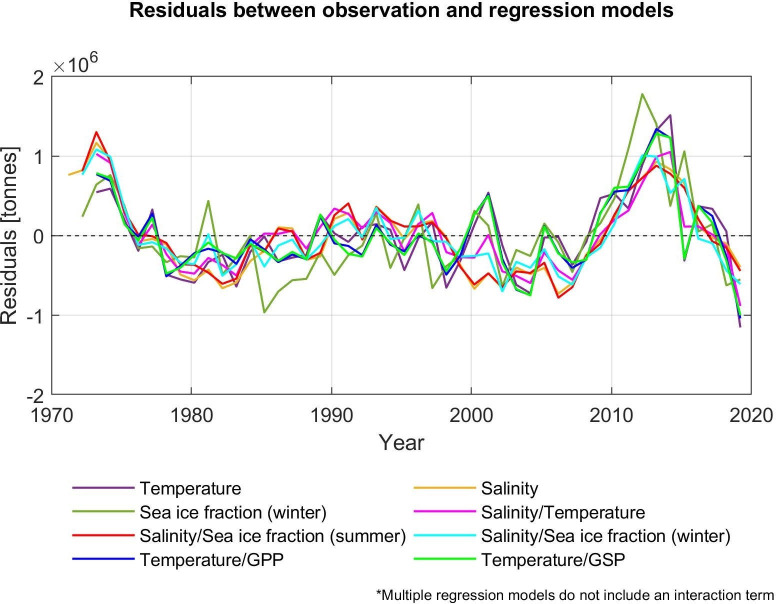
TSB difference between observations and simple/multiple regression models. Residuals are calculated from subtraction between observations and each regression model (0.5 > *R*^2^). For simplicity, only multiple regression models without an interaction term are plotted.

Our regression models fit well before 2010, but show a decline in TSB from 2010 while diverging from the observations that reached the historical highest level in 2013 (Fig 7). There are large errors around 2012–2015 at approximately 0.7–1.7 × 10^6^ tonnes in several models while differences between models and observations vary between around −1.0 × 10^6^ and 0.5 × 10^6^ tonnes in all models in the preceding 40 years (Fig 8). Since 2010, the mismatch between the observations and our models has prolonged.

The AIC of the multiple regression models (the number of parameters: *m* = 3 or 4; Table 2) is little smaller than that of the simple regression models (*m* = 2; Table 2). When comparing two different types of multiple regression models, the AIC of the multiple regression models with an interaction term (No. 2–1, 2–3, 2–5, 2–7, 2–9; *m* = 4) is little higher than that of the multiple regression models without an interaction term (No. 2–2, 2–4, 2–6, 2–8, 2–10; *m* = 3) although the *R*^2^ values of these models are almost the same, suggesting that the regression model with two explanatory variables is the better-fit model. The regression coefficients of slope (*α*_1_, *α*_2_) of several regression models are significantly different from zero (S4 and S5 Tables). On the other hand, the interaction terms of all multiple regression models are not important because they do not explain the variations of TSB (*p* > 0.05; S5 Table).

Based on the rule of multicollinearity, two variables in the multiple regression model No. 2–5 (salinity/winter sea ice), salinity and an interaction term (salinity × sea ice concentration in winter), have high *VIF* (S5 Table), and these two variables are strongly related to each other (*r* = 0.97; S6 Table), so that the model No. 2–5 will not be selected as the best regression model for TSB. With the exception of this model, explanatory variables in multiple regression models have lower *VIF* (*VIF* < 3.0; S5 Table), and variables are not strongly correlated each other (*r* < 0.90; S6 Table).

In conclusion, the model No. 2–2 (based on temperature/salinity), 2–6 (salinity/sea ice concentration in winter), 2–8, (temperature/GPP) and 2–10 (temperature/GSP) are defined as the statistical best-fit models to TSB. The equations of best-fit models to TSB are given as

Model No.2−2:


$${TSB}_{a}=1.1\times 10^{7}\times {Salinity}_{a-1}+7.9\times 10^{5}\times {Temperature}_{a-3}+2.1\times 10^{4}$$

Model No.2–6:


$${TSB}_{a}=1.2\times 10^{7}\times {Salinity}_{a-1}-2.3\times 10^{6}\times {Sea\;ice\;concentration}_{a-2}+9.9\times 10^{5}$$

Model No.2–8:


$${TSB}_{a}=1.4\times 10^{6}\times {Temperature}_{a-3}+1.1\times 10^{4}\times {GPP}_{a-2}+7.3\times 10^{4}$$

Model No.2–10:


$${TSB}_{a}=1.4\times 10^{6}\times {Temperature}_{a-3}+2.2\times 10^{4}\times {GSP}_{a-2}+7.2\times 10^{4}$$

### Application of regression models to downscaled climate projections

**Fig 9:**
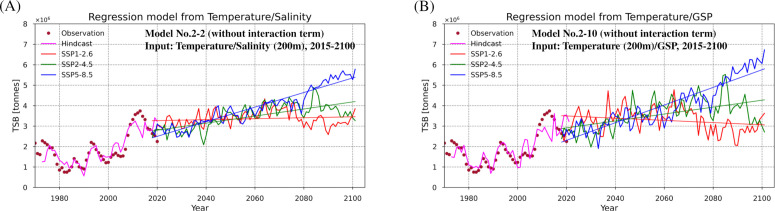
Projection of TSB estimated with regression models using variables with future climate scenarios. TSB estimated with regression models using variables with future climate scenarios: SSP1-2.6 (red lines), SSP2-4.5 (green lines), SSP5-8.5 (blue lines), observations (spots) and TSB predicted from hindcast (pink lines) are shown. TSB is calculated from **(A)** temperature and salinity with regression model No. 2-2 (without an interaction term), and **(B)** temperature and gross secondary production with regression model No. 2-10 (without an interaction term). Note that bias correction for temperature, salinity, GSP in projections is employed (see “Application of regression models to downscaled climate projections” in the “Discussion” for more details on bias correction).

Based on the evaluation of the regression models (Table 2), we choose seven regression models based on variables in the Barents Sea, which have high prediction skill (high *R*^2^ and *p* < 0.01). Then, we apply these to each of the three climate scenarios (SSP1–2.6, SSP2–4.5, SSP5–8.5) for the period 2015−2100 (Fig 9 and S8 Fig). Note that bias correction is employed for all variables by shifting a constant value from the projections (salinity: −0.25psu, temperature: + 1°C, GSP: + 15 gCm^-2^ yr^-1^) because there are biases for explanatory variables in the projections.

In general, TSB has a positive trend in the SSP5–8.5 scenario, whereas it has a negative trend in the SSP1–2.6 scenario except for when future TSB is estimated based on salinity (No. 1-2-1; S8 Fig B). When TSB is estimated with the simple/multiple regression models based on temperature, strong positive trends are apparent in SSP5–8.5 (Fig 9A, Fig 9B, S8 Fig A, S8 Fig C, S8 Fig E). In contrast, there is a less positive trend in TSB when TSB is estimated with the simple regression model based on GSP in SSP5–8.5 (S8 Fig D). For SSP1–2.6, large negative trends in TSB are shown when TSB is estimated with temperature-based model (S8 Fig A) or the multiple regression models based on temperature and GSP (Fig 9B, S8 Fig E) while there is a less negative trend in TSB with GSP-based model (S8 Fig D).

## Discussion

There is little doubt that knowledge about the future status of the NEA cod stock can give valuable information for fisheries scientists, fisheries and environmental managers, resource economists, policy experts, managers, the fishing industry, and investors in the fishing fleet. However, the big question is how feasible it is to make reliable and appliable predictions and projections.

A key objective within fish stock biomass predictability is to determine what the main sources of predictive robustness are, given the lead time of interest and the population’s life history. Main sources contributing to precision for fish biomass predictions are reliable forecasts of recruit abundance (young fish surviving the first vulnerable life stages) and individual growth (weight gain) through life. In addition, for fished stocks, biomass removed by fisheries may constitute a major fraction of the total biomass. For some fish stocks, not least sub-arctic populations like Northeast Arctic cod, interannual variability in both recruitment and individual growth is linked to environmental variability (as pointed to already by [1,7]). These connections are the basis for our study, where we conduct both short-term predictions and projections of the total stock biomass (TSB) of the NEA cod by means of statistical modelling.

We develop simple and multiple linear regression models based on gridded variables from the regional ocean model NEMO-NAA10km and the ecosystem model NORWECOM.E2E. A wide range of environmental variables, temperature, salinity, sea ice concentration, gross primary production and/or gross secondary production, are here used to develop statistical models trained against historical TSB values from ICES stock assessments.

We find that several of our regression models have high prediction skill and capture the variations in TSB well, in particular, the regression models based on temperature. This is most likely because TSB is strongly influenced by the variations in temperature both directly and indirectly in different ways. Increased ocean temperature has been shown to positively affect growth rate [54,55] and length of cod [55,56], and thereby also recruitment [55]. Reduced sea ice extent owing to high ocean temperature [16] may result in an increased suitable feeding area for cod, leading to a large cod stock [28]. Moreover, cod stock biomass may increase with high abundance of copepods, the main prey of the early stages of cod [25,34]. TSB therefore has an increasing trend with an increase in prey abundance (GSP) in the warming scenario (SSP5–8.5), while it has a negative trend in the low emission scenario (SSP1–2.6) (Fig 9).

Our approach is in part similar to that of Årthun et al. (2018) [7] and Koul et al. (2021) [32] in that both these papers applied linear regression models. However, they did not include any type of prey. A major step forward is that we in our models also examine the predictive capacity of primary and secondary production, provided by NORWECOM.E2E. Another significant expansion is that we also include projections from now and towards the end of the twenty-first century within a range of IPCC type climate scenarios.

There are, admittedly, important factors that are not included in our predictions and projections, notably the variability in fishing pressure over time. The challenge here is that human behavior and decisions set the premises for the factors determining fishing pressure, such as annual fishing quotas, longer term harvest control rules and many other stock regulation rules. To our knowledge, no models can reliably predict such human behavior. Even large full population models still only assume different future levels of fishing pressures based on some predetermined rules that managers will or will not follow or change. Fishing technology is also in continuous development, making fisheries more effective and easier to target specific age groups/sizes. In this study we have chosen not to try to predict future fishing mortality or technology. It can be argued, as done by Årthun et al. (2018) [7], that present fishing mortality and technology implicitly already is taken into account in the stock estimates from the ICES stock assessment. Therefore, assuming that these will not change too much in the future, our approach can be looked at as assuming retained status quo with regards to human influence. Our results should therefore be interpreted as a bottom-up approach, examining how much future values of key environmental factors alone can explain future stock development.

Further, abiotic and lower trophic level variables are not the only environmental factors determining the NEA cod stock development. The cod does not live in isolation, but interacts not only with plankton, but other fish species, marine mammals and seabirds. However, when moving further into the future than the 1–3 years that is typical in stock assessment today, there is a lack of (spatially resolved) higher trophic level predictions or projections (although see Nilsen et al. (2025) [57] applying the NoBa Atlantis model).

Therefore, our approach is based on the time series that are available from downscaled physical and lower trophic level ecosystem models. In the future, when, hopefully, more stock and ecosystem data for the future are available, regressions should be expanded to include these, as well as utilizing more advanced statistical approaches. Until then we must use the methods available and learn what we can from them.

The total impact of change in temperature and access to prey on the cod stock biomass will vary between the scenarios depending on how optimal the future temperature will be compared to the specific growth rate at different life stages and how much its main prey will be impacted. The sensitivity analysis for capelin in Sandø et al. (2024) [36], performed with the same ocean and ecosystem model results as in this study, indicates that the total impact of change in temperature, sea ice extent, and prey in terms of plankton production is considered to be small in SSP1–2.6, moderately positive in SSP2–4.5, and correspondingly negative in SSP5–8.5. As such a relationship between climate, capelin and NEA cod is not implemented in our ecosystem model, it is reasonable to assume that our cod projections in SSP5–8.5 could be overly optimistic.

Except from experimental studies in laboratories and knowledge about thriving ranges of different environmental variables such as temperature, there is limited knowledge about how environmental variables will affect the marine ecosystem in the future. Though, the available information is implemented in ecosystem models so that sensitivity studies on primary and secondary production can be performed. Results from a study on controlling factors of Barents Sea plankton production in a fluctuating climate showed only minor changes in the distribution between diatoms and flagellates, and between micro- and mesoplankton during a scenario representing intermediate emissions of greenhouse gasses [41].

Due to highly variable knowledge about how different climate exposures affect higher trophic species, and thereby lack of higher trophic level model results, it is hard to model how different species will affect each other in the future [36]. Climate vulnerability studies are therefore often based on climate exposures consisting of physical and biogeochemical variables, and sensitivity attributes such as habitat and prey specificity at different life stages [36,58,59].

That said, the uncertainties with such sensitivity analyses in terms of climate exposures from model results are not insignificant [36], and are among other things related to model errors, internal variability in the climate system, varying knowledge about how different species respond to external influences, interaction between species, and last, but not least, future fishing pressure.

In the following, we discuss our predictions and projections in terms of the correlation and regression analyses from Results in more detail.

### Lag correlation analysis

According to the lag correlation analysis, high correlations between TSB and hydrographic and biological time series are found at several locations. Focusing on the correlation between TSB and salinity time series at 200m depth, the time lags decrease as latitude increases toward the Arctic along the pathway of NAC/NwAC (Table 1, Fig 6C, Fig 6D); a high correlation between TSB and salinity at the Faroe-Shetland Channel (FSC) with a lag of 6 years, in the Norwegian Sea with a lag of 5 years, at BSO with a lag of 3 years, in the Barents Sea with a lag of 1 year. These results are similar to those of Årthun et al. (2018) [7] who showed that variations in salinity at the Greenland–Scotland ridge (FSC/IFR) lead to variations in the Barents Sea salinity after 2 years, and to variations in TSB after 7 years. Similarly, considering the correlation between TSB and temperature time series at 200 m depth, the time lags also decrease towards the Arctic along the Atlantic inflow; a high correlation between TSB and temperature at the Rockall Trough (RT) with a time lag of 6 years, in the southern Norwegian Sea (NwSS) with a lag of 4 years, at BSO with a lag of 3 years (Table 1, Fig 6B). This supports to some extent the earlier study, which reported that high temperatures at BSO correspond to high cod biomass with a lag of 2 years [7]. However, when we consider the correlation between TSB and temperature time series at FSC, there are two peaks of correlations; one at 0–1 year lag and one at 5–6 years lag (Fig 6B). This is also found if we consider sea surface temperature and salinity (S9 Fig). Although it is expected that there will be a high correlation between TSB and temperature at 200 m depth at FSC at a lag of around 7 years [7], the correlation is somewhat lower at 5–6 years lag than that at 0–1 year lag. The shortest time lag is probably due to a large-scale synoptic response to the atmosphere [60,61] that is transferred more or less instantaneously by vertical mixing to depths of 200 m, while the longer time lag is due to advection of Atlantic Water [62]. A higher correlation at 0–1 year than at 5–6 years might be an indication of less preserved temperatures at 200 m due to too strong vertical mixing, and thereby a dilution of the advective signal. This needs to be investigated further.

In agreement with Årthun et al. (2018) [7], there is a high correlation between TSB and AMO index with a lag of 6 years (Table 1, Fig 6A). Winter sea ice concentration in the Barents Sea is highly correlated with TSB with a lag of 1–2 years (Table 1, Fig 6F) while correlation between TSB and summer sea ice concentration is quite low (S1 Table, Fig 6E). The sea ice extent in summer (Fig 3E, S3 Fig) has been low in the Barents Sea (average sea ice concentration of less than 10%) for four decades, so that it may not be a suitable explanatory variable in the regression model. Focusing on the biological variables, there is a high correlation between TSB and GPP time series in the whole Barents Sea (BS) and southern Barents Sea (BSS) with a lag of 2 years, but with a lag of 3 years in the northern Barents Sea (BSN) (Table 1, Fig 6G). In contrast, there is no correlation between TSB and upstream phytoplankton biomass at the Greenland–Scotland ridge (FSC and IFR) and in the Norwegian Sea (NwS) (S1 Table, Fig 6G, Fig 6H). This suggests that TSB is influenced largely by the local variability of primary production.

There is also a high correlation between TSB and GSP time series in the Barents Sea with a lag of 2 years (also in the northern and southern Barents Sea; Table 1, Fig 6I). Although the correlation between TSB and upstream zooplankton biomass is low (at FSC and IFR; S1 Table), there is a high correlation between TSB and GSP in the Norwegian Sea (NwS, NwSN, NwSS) with a lag of 6–9 years (Table 1, Fig 6I, Fig 6J). This suggests that TSB can be affected by both local and neighboring variability of secondary production. Zooplankton is transported by the NwAC from the Norwegian Sea into the Barents Sea along the coast [30], and *Calanus finmarchicus* is dominating in both the Norwegian Sea and the Barents Sea [20]. In addition, the NEA cod spawns along the Norwegian coast and their larvae drifts into the Barents Sea which is their nursery area [20]. The first food of the NEA cod larvae is nauplii of copepods, and the main prey of larger larvae is copepodites of *C. finmarchicus* [25]. Additionally, as cod increases in size, there is a shift in diet towards lager prey items such as krill and fish (e.g., capelin) [34]. Capelin is also a main planktivorous predator in the Barents Sea ecosystem [63], and is a major food item for mature cod [20,64]. Therefore, food competition between the two species and predation by cod may impact capelin stock. Therefore, the result in this study suggests that TSB increases with abundant copepods in both the Norwegian Sea and the Barents Sea. The large time lags identified here must, however, be interpreted with some care as primary and secondary production is very dependent on the large scale atmospheric physical fields and associated vertical mixing of nutrients [41]. Changes in such large-scale physical fields can be very dominant in the relatively small locations along the rim of the deep Norwegian Sea (Fig 1), and the GSP in the relatively small NwS location may therefore represent long-term variability that is much bigger than in the ocean basin itself [65].

### Regression analysis and predictions

TSB has a high correlation with almost all investigated explanatory variables in the Barents Sea, with a lag of 1–3 years (Table 1), and the multiple regression models presented here can predict variation in TSB 1–2 years in advance (Table 2). For instance, the model No. 2–1 (temperature/salinity) can predict TSB 1 year in advance because salinity leads TSB by 1 year even if temperature leads TSB by 3 years. Hence, the prediction horizon depends on the explanatory variable with the shortest time lag. The prediction horizon of our multiple regression models is 5–6 years shorter than that of the regression models presented in a previous study by Årthun et al. (2018) [7]. This is because they used the upstream hydrographic variables (e.g., temperature, salinity at the Faroe-Shetland Channel) that lead TSB by 7 years whereas we use the local hydrographic/biological variables (e.g., temperature, salinity, GSP in the Barents Sea) that lead TSB by 1–3 years as predictors. In other words, the prediction horizon of our multiple regression models is likely to be longer if the models are based on upstream variables along the NAC/NwAC. In fact, the prediction horizon of our simple regression models is much longer if the upstream variables are used as explanatory variables (e.g., No. 1-1-10 model, based on salinity at the Rockall Trough (RT), can capture 55% of TSB variation 6 years in advance; Table 2, S6 Fig D). However, the cost for longer prediction horizon is a lower prediction skill. The shorter time lags would be expected to be better for predictions, as the network of indirect effect linking the explanatory variables and cod biomass is necessarily much simpler.

### Evaluation of regression models

When we focus on the simple regression models, which are constructed only by variables in the Barents Sea, the prediction skill is high (*R*^2^ > 0.56; Table 2) if the regression model includes temperature, salinity, or sea ice concentration in winter in the Barents Sea as an explanatory variable. Moreover, the simple linear regression models based on biological variables (GPP or GSP) also have good prediction skills (*R*^2^ ≥ 0.45; Table 2). Meanwhile, the multiple regression models fit better in terms of AIC values (more than 2 points lower than the simple regression models; Table 2).

As the AIC shows (Table 2), the two-predictor multiple regression model without interactions (the number of parameters: *m* = 3) fits better than those with interactions (*m* = 4). When we focus on the multiple regression models without interactions (*m* = 3), the model No. 2−2 (based on temperature/salinity) can be the best model for TSB because this model has a minimum value of AIC (Table 2). Moreover, we consider that the model No. 2−6 (salinity/sea ice concentration in winter, without interactions) is the best model for TSB because it has higher *R*^2^ (*R*^2^ = 0.72; Table 2). As we mentioned above, high ocean temperature causes reduced sea ice extent [16], and it may result in an increased suitable feeding area for cod and it will lead to a large cod stock [28]. Therefore, the model No. 2−6 should not be ignored even though the value of delta AIC is higher (delta AIC = 33.13; Table 2). The model No. 2−8 (temperature/GPP: delta AIC = 10.48) and No. 2−10 (temperature/GSP: delta AIC = 9.53) are also selected as the best-fit models even though the delta AICs are little higher compared to the No. 2−2 because these models are based on biological variables and should not be ignored [50]. Additionally, the interaction terms of all multiple regression models are not important (*p* > 0.05; S5 Table). These results would suggest that two variables (e.g., temperature and salinity) have separate effects on biomass and do not have interactive or synergistic effects.

In conclusion, the model No. 2–2 (based on temperature/salinity), 2–6 (salinity/sea ice concentration in winter), 2–8, (temperature/GPP) and 2–10 (temperature/GSP) are selected as the statistical best-fit models to TSB.

In general, the ability of nutrients-phytoplankton-zooplankton (NPZ) models to reproduce spatial and temporal variations will depend on various factors, including model complexity, parameterization, resolution, and the availability of observational data for validation. NORWECOM.E2E is tailored for high-latitude ecosystems, such as the Norwegian continental shelf and Arctic environments, which exhibit strong seasonal variation in light, nutrients and biological productivity [39]. Misrepresentations of the mixed layer depth in global models may have strong consequences for the for the primary production [66] and may lead to match-mismatch problems related to primary and secondary production in the ecosystem model [67]. However, increased model resolution and downscaling will tend to reduce the uncertainty due to better representation of the circulation, hydrography, position of the sea ice edge and last, but not least, the timing of the spring bloom as shown in Skogen et al. (2018) [40]. Despite improved physics from downscaling, estimations of primary and secondary production from NPZ models are apparently not yet sufficiently accurate and may therefore contribute to the poorer performance of models No. 2−8 and No. 2−10 in comparison with No. 2−2.

### Interpretation of regression models

As indicated above, four multiple regression models (No. 2–2, 2–6, 2–8, and 2–10) are defined as the best-fit models to TSB. These models are based on variables that affect cod directly and indirectly, thus our statistical models show the comprehensive relationship between TSB and these variables. First, three out of four suited multiple regression models (No. 2–2, 2–8, and 2–10) include temperature in the Barents Sea as an explanatory variable. Temperature is one of the most important environmental factors for the cod stock and affects aspects of the cod population in several ways. For instance, ocean temperature has a positive impact on growth rate [54,55] and length of cod [55,56], resulting in a positive effect on recruitment [55]. Furthermore, sea ice extent is reduced owing to high ocean temperature [16] and increased temperature is therefore positively linked to the suitable feeding area, leading to a large cod stock [28]. Moreover, reduced sea ice concentration and expanded open-water area is positively associated with increased primary production [35,68,69], and zooplankton biomass may therefore increase when feeding on these increased phytoplankton blooms [20].

Furthermore, Sandø et al. (2021) [41] found that there was a strong relationship between plankton production and climate-related factors such as heat transport by Atlantic water through the BSO, ocean temperature, light, sea ice concentration, vertical mixing of nutrients, which all covaried with the atmospheric forcing. Skjoldal et al. (1986) and Skjoldal and Rey (1989) [70,71] linked the variations in the zooplankton abundance in the western Barents Sea to the variations in the inflow of zooplankton-rich Atlantic water from the Norwegian Sea. *C. finmarchicus* is the major prey species for larvae cod and juveniles, and cod stock biomass may increase with abundant copepods in both the Norwegian Sea and the Barents Sea [25,34]. This chain of events illustrates how variability in temperature and transports can be linked to variability in plankton abundance and TSB.

### Application of regression models to downscaled climate projections

The mean temperature in the Barents Sea increases about 2°C by the year 2100 in SSP5–8.5 scenario (Fig 5A). Fig 9A and Fig 9B (also S8 Fig A, S8 Fig C and S8 Fig E) show that there is a pronounced positive trend of TSB in SSP5–8.5 scenario when TSB is estimated by using simple regression models based on temperature only, or multiple regression models based on the combination of temperature and salinity/GSP. This result is consistent with [72] who reported that cod stock increases at temperature increments of 1 or 2°C. In contrast, there is a negative trend of TSB in SSP1–2.6 (Fig 9B, S8 Fig A, S8 Fig D, S8 Fig E). This negative trend of TSB is due to a negative temperature trend as well as a negative trend of GSP in SSP1–2.6 (Fig 5A, Fig 5C).

Since there are biases for explanatory variables in the projections, the bias correction is employed for all variables by shifting a constant value from the projections before the regression models are applied to downscaled climate projections. In particular, there are large salinity biases between the hindcast simulation and the projections (with salinities of approximately 35 psu in the last year of hindcast and 35.3 psu in the first year of projections; S10 Fig A). This is because the hindcast simulation is forced by a realistic atmospheric forcing while the future projections are downscaled from a coupled global climate model. This will normally lead to differences between the observations and simulations during the overlapping period due to different phases of natural variability. In addition, both the global and regional models have their own biases due to model imperfectness. To make these simulations comparable, bias correction for salinity is employed, removing 0.25 psu from the projections in all scenarios throughout the whole year (S10 Fig B). After the bias correction, the same regression model, based on salinity (No. 1-2-1), is used to project TSB. S11 Fig A shows a comparison of TSB from observations, TSB predicted from the hindcast simulation and TSB projected from future climate scenarios, and S11 Fig B also shows a comparison of TSB, but where the bias correction for salinity is employed in the projections. After the bias correction, TSB ranges approximately 2–5 million tonnes, and is of the same order as the observations between 1946–2020 (Fig 2). Therefore, it is likely that the regression models can project TSB after the bias correction. There are different methods to correct biases in climate model simulations [73]. In this study, one of the simplest bias correction methods is used, shifting a constant value from the projections. This method assumes that model biases remain constant in the projections. However, further study of evaluation and comparison of bias correction methods is outside the study’s scope.

### Further improvement of regression models and perspectives

The main objective of this study is (was) firstly to explore how well simple statistical modeling combined with essential environmental variables time series from dynamic modeling can estimate the total cod biomass, and secondly to apply different regression models to long-term projections under the assumption that nothing else will change in the ecosystem. These projections do not include future fishing pressure, not because we don’t think it is important, but simply because we don’t have projections for it. Changes in fishing pressure have happened in the past due to variability in the total cod biomass, and changes will come. Moreover, fishing pressure is highly influenced by the future behavior of the fishing fleet, development of fishing technology and management regulations. All these human behavior related factors have influenced the historic development of the fish stock, and will undoubtedly continue to do so, but they are also unfortunately close to impossible to predict (project) on longer time scales.

In Fig 8, there are large errors between observations and regression models around 2012–2015 for the TSB. These errors are also shown in Årthun et al. (2018) [7] and their models also underestimated cod biomass around 2014. Although a harvest control rule that determines the total allowable catch for cod was introduced in 2004 [74–76], resulting in higher TSB after implementation [28], the prediction models do not include changes in fishing pressure. As a result, the predictions by statistical models lead to an underestimation of cod biomass [7]. Indeed, Årthun et al. (2018) [7] argued that the underestimated predictions after 2010 are consistent with lower harvest rates after 2007. Therefore, as also highlighted by Årthun et al. (2018) [7], fishing pressure scenarios should be taken into consideration when the prediction model is developed in future studies. One approach to this problem is to include fishing pressure in the models under the assumption that the current “average” fishing pressure will remain in the future. Another approach is to include different ﬁshing scenarios which were used in Koul et al. (2021) [32].

Our regression models which include biological variables do not always show a significant improvement. The prediction skill of the multiple regression model based on temperature and gross secondary prediction (GSP) is little higher (*R*^2^ = 0.69; Table 2) than the simple regression models based on temperature only (*R*^2^ = 0.65; Table 2). However, the regression model based on temperature and salinity (*R*^2^ = 0.75; Table 2) fits better than the regression model based on temperature and GSP (*R*^2^ = 0.69; Table 2). Moreover, the prediction skill of our model based on temperature and GSP is lower (*R*^2^ = 0.69; Table 2) than the model based on sea surface temperature and ﬁshing mortality which was developed by Koul et al. (2021) (*R*^2^ = 0.84, [32]). This suggests that other variables should be also included. First, as we have discussed above, our models do not include changes in fishing pressure. This may lead to the mismatch between the observations and predictions of cod biomass. Second, food availability is also an important aspect to predict/project cod biomass. Our regression models include biological variables, such as GSP because zooplankton, especially copepod, is the main prey of the early stages of cod. However, our models do not include other prey items for adult cod, in particular the capelin (*Mallotus villosus*), which is a major food source for mature cod [20,64] and capelin availability can be an important driver of cod stock [77]. Cod biomass can be also influenced by other prey species. Therefore, other factors such as fishing pressure and other prey items for cod (e.g., capelin) should be included in the models. However, further development of prediction models is beyond the scope of this study.

Extending the multiple regression models with other variables could potentially improve the future projections of cod biomass. In this study, multiple regression models have been developed with only two variables since we want to minimize the number of explanatory variables and prevent overfitting of the models. Further studies are required to assess the predictability of multiple regression models with three or more explanatory variables.

There are a range of different alternatives to the linear regression model approach we have chosen. More general approaches are available in the model classes of Generalized Linear Models (GLMs), which allows the linear model to be related to the response variable via a link function [78] and Generalized Additive Models (GAMs) where the linear response variable depends linearly on unknown smooth functions of some predictor variables [79]. GLMs and especially GAMs, with the added freedom allowed, likely provide better explanatory power for an existing (or training) data set. Unfortunately, their usefulness for prediction/forecasting is at best debated as their smooth functions may provide unstable predictions outside the range of existing data [80]. Further, simplicity is important in explorative analyses like ours, especially when testing the explanatory power of a range of different variables. In addition, Årthun et al. (2018) [7] and Koul et al. (2021) [32] were quite successful when they predicted cod biomass using linear regression models and our intent is to build upon these earlier works.

## Conclusions

This study aims to explore how well simple statistical modeling can be applied to short-term predictions and long-term projections of the biomass of the NEA cod in the Barents Sea. We examine the predictability of statistical models only based on hydrographic and lower trophic level biological variables from dynamical modeling. Simple and multiple linear regression models are developed based on gridded variables from the regional ocean model NEMO-NAA10km and the ecosystem model NORWECOM.E2E. The regression models are statistically evaluated to find variables that can capture variability in Barents Sea cod biomass. Finally, future TSB is projected by applying the best regression models to the range of downscaled IPCC CMIP6 climate scenarios; SSP1–2.6, SSP2–4.5, SSP5–8.5. Our prediction models are based on variables that affect cod both directly and indirectly.

We find that several of our suggested regression models have high prediction skill and capture the variations in TSB well, in particular, the regression models based on temperature. This is most likely because TSB is strongly influenced by the variations in temperature both directly and indirectly in different ways. Our results suggest that increased ocean temperature and abundant zooplankton may lead to a large cod stock. However, even if total stock biomass has a positive trend with an increase in copepods in the highest warming scenario (SSP5–8.5), we found that it has a negative trend in the low emission scenario (SSP1–2.6) when the regional ocean and ecosystem models show weak cooling and reduced zooplankton.

Investigating the impact of climate variability and change on fish stock in terms of statistical and/or ecosystem models can be valuable for future studies. We show that variability in essential environmental variables can provide a remarkably good first approximation to cod dynamics. However, further improvement of our regression models is needed because variations in TSB are not estimated precisely. To resolve the full picture, other factors like fishing and natural mortality also need to be addressed explicitly.

## Supporting information

S1 Table Lag correlation analysis between TSB and hydrographic/biological time series in the Barents Sea/Norwegian Sea, along the NAC/NwAC, AMO index. (PDF)

S2 Table List of simple regression models for total stock biomass of the NEA cod in the Barents Sea (TSB) and statistics of the regression models. (PDF)

S3 Table List of multiple regression models for total stock biomass of the NEA cod in the Barents Sea (TSB) and statistics of the regression models. (PDF)

S4 Table Statistics of the regression coefficients of the simple regression models. (PDF)

S5 Table Statistics of the regression coefficients of the multiple regression models. (PDF)

S6 Table Correlations of variables used in the multiple regression models. (PDF)

S1 Fig Maps of temperature at 200m depth. Two representative maps (A) in 1970, and (B) in 2019 are given. Data is also available between 1971–2018.
(TIF)

S2 Fig Maps of salinity at 200m depth. Two representative maps (A) in 1970, and (B) in 2019 are given. Data is also available between 1971–2018.
(TIF)

S3 Fig Maps of Barents Sea and Norwegian Sea ice concentration. Representative maps are given; (A) maximum ice cover in 1979 in winter, and (B) minimum ice cover in 2016 in winter during recent years, (C) ice cover in 1979 in summer, (D) ice cover in 2016 in summer. Data is also available between 1970–2019.
(TIF)

S4 Fig Maps of gross primary production at surface. Two representative maps (A) in 1970, and (B) in 2019 are given. Data is also available between 1971–2018.
(TIF)

S5 Fig Maps of gross secondary production at surface. Two representative maps (A) in 1970, and (B) in 2019 are given. Data is also available between 1971–2018.
(TIF)

S6 Fig Comparison between observations and simple regression models. In each figure, spots show the total stock biomass of the NEA cod (TSB), and solid line shows TSB estimated by regression models. Each regression model is constructed by variables below: (A) temperature at 200m depth in the Barents Sea (BS) and the Norwegian Sea (NwS), and AMO index (B) temperature along the NAC/NwAC, (C) salinity at 200m depth at BS and NwS, (D) salinity along the NAC/NwAC, (E) sea ice fraction in summer at BS and NwS, (F) sea ice fraction in winter at BS and NwS, (G) GPP at BS and NwS (H) GPP along the NAC/NwAC, (I) GSP at BS and NwS, and (J) GSP along the NAC/NwAC. Anomalies are relative to 1970–2019. Abbreviations of focus area and variable names are defined in Fig 1.
(TIF)

S7 Fig Comparison between observations and multiple regression models. In each figure, spots show the total stock biomass of the NEA cod (TSB), and solid line shows TSB estimated by multiple regression models. Each regression model is constructed by two variables, which are obtained in the Barents Sea. There are two types of multiple regression models: (A) one includes an interaction term, and (B) another has no interaction term. Anomalies are relative to 1970–2019.
(TIF)

S8 Fig Projection of TSB estimated with regression models using variables with future climate scenarios. TSB estimated with regression models using variables with future climate scenarios: SSP1–2.6 (red lines), SSP2–4.5 (green lines), SSP5–8.5 (blue lines), observations (spots) and TSB predicted from hindcast (pink lines) are shown. TSB is calculated from (A) temperature with regression model No.1-1-1, (B) salinity with regression model No. 1-2-1, (C) temperature and salinity with regression model No. 2–1 (with an interaction term), (D) gross secondary production with regression model No. 1-6-1, (E) temperature and gross secondary production with regression model No. 2–9 (with an interaction term). Note that bias correction for temperature, salinity, GSP in projections is employed (see “Application of regression models to downscaled climate projections” in the “Discussion” for more details on bias correction).
(TIF)

S9 Fig Lag correlation analysis between total stock biomass (TSB) and temperature/salinity at surface. Cross correlations between TSB and (A) temperature at surface in the Barents Sea (BS) and the Norwegian Sea (NwS), (B) temperature along the NAC/NwAC, (C) salinity at surface in the BS and NwS, (D) salinity along the NAC/NwAC, are shown. The spots show the maximum correlation. Abbreviations of focus area names are defined in Fig 1.
(TIF)

S10 Fig Time series of salinity in the Barents Sea. (A) Time series of salinity at 200 m depth in the Barents Sea (original data). Salinity from hindcast simulation (pink line), and from projections (red, green, blue) are shown. (B) A bias correction for salinity in projections is employed (salinity – 0.25 psu).
(TIF)

S11 Fig Comparison of TSB estimated from regression model. (A) Comparison of TSB estimated from regression model No. 1-2-1, based on salinity. Observation (spots), TSB predicted from hindcast simulation (pink line), and TSB projected from future climate scenarios (red, green, blue lines) are shown. (B) A bias correction for salinity in projections is employed (salinity – 0.25 psu).
(TIF)

S1 File Supplementary text. (PDF)
