# Supplementary material for: Exploration of short-term predictions and long-term projections of Barents Sea cod biomass using statistical methods on data from dynamical models
Source: PLoS One. 2025 Jul 31;20(7):e0328762. doi: 10.1371/journal.pone.0328762 (PMC12312909; doi:10.1371/journal.pone.0328762)
Supplement: S1 Table — (PDF) [file pone.0328762.s001.pdf]

S1 Table. Lag correlation analysis between TSB and hydrographic/biological time series in the Barents Sea/Norwegian Sea, along the NAC/NwAC, AMO index.

Focus area	Temperature at 200m depth			Salinity at 200m depth		
	r	*p*-value	Time lag [year(s)]	*r*	*p*-value	Time lag [year(s)]
BS	0.80	$p < 0.001$	3	0.81	$p < 0.001$	1
BSN	0.62	$p < 0.001$	4	0.73	$p < 0.001$	1
BSS	0.77	$p < 0.001$	3	0.79	$p < 0.001$	1
NwS	0.50	$p < 0.001$	0	0.78	$p < 0.001$	5
FSC	0.50	$p < 0.001$	0	0.62	$p < 0.001$	6
IFR	0.08	5.87E-01	0	0.23	1.05E-01	0
BSO	0.76	$p < 0.001$	3	0.82	$p < 0.001$	3
NwSN	0.61	$p < 0.001$	1	0.75	$p < 0.001$	7
NwSS	0.64	$p < 0.001$	4	0.80	$p < 0.001$	6
RT	0.74	$p < 0.001$	6	0.80	$p < 0.001$	6

Focus area	Sea ice concentration in summer			Sea ice concentration in winter		
	r	*p*-value	Time lag [year(s)]	*r*	*p*-value	Time lag [year(s)]
BS	-0.32	2.51E-02	2	-0.75	$p < 0.001$	2
BSN	-0.32	2.53E-02	2	-0.73	$p < 0.001$	2
BSS	-0.33	2.50E-02	4	-0.59	$p < 0.001$	1
NwS	-0.21	1.54E-01	1	-0.59	$p < 0.001$	2

Focus area	Gross primary production (GPP)			Gross seconday production (GSP)		
	r	*p*-value	Time lag [year(s)]	*r*	*p*-value	Time lag [year(s)]
BS	0.67	$p < 0.001$	2	0.67	$p < 0.001$	2
BSN	0.63	$p < 0.001$	3	0.65	$p < 0.001$	3
BSS	0.60	$p < 0.001$	2	0.62	$p < 0.001$	2
NwS	0.05	7.63E-01	5	0.73	$p < 0.001$	6
FSC	0.29	5.57E-02	6	0.21	1.78E-01	6
IFR	0.25	9.68E-02	5	0.30	3.50E-02	1
BSO	0.46	2.00E-03	8	0.52	$p < 0.001$	8
NwSN	0.09	5.46E-01	5	0.74	$p < 0.001$	7
NwSS	-0.03	8.54E-01	5	0.56	$p < 0.001$	9

AMO index		
r	*p*-value	Time lag [year(s)]
0.71	$p < 0.001$	6

Maximum correlation coefficient (*r*) between TSB and variable, *p*-value and time lag which has maximum correlation are given. Note that time series of GPP and GSP at Rockall Trough (RT) are not available because this focus area is located outside of the ecosystem model domain. Abbreviations of focus area names are defined in S7 Table.
