# Supplementary material for: Exploration of short-term predictions and long-term projections of Barents Sea cod biomass using statistical methods on data from dynamical models
Source: PLoS One. 2025 Jul 31;20(7):e0328762. doi: 10.1371/journal.pone.0328762 (PMC12312909; doi:10.1371/journal.pone.0328762)
Supplement: S2 Table — (PDF) [file pone.0328762.s002.pdf]

S2 Table. List of simple regression models for total stock biomass of the NEA cod in the Barents Sea (TSB) and statistics of the regression models.

Model No.	Variable(s)		Station	R^2	*F*-statistics	DF	*p*-value	*RSS*	AIC
	x_1	Time lag							
1-1-1	Temperature (200 m)	3	BS	0.65	8.25E+01	1 and 45	$p < 0.001$	1.19E+13	1374.63
1-1-2	Temperature (200 m)	4	BSN	0.39	2.75E+01	1 and 44	$p < 0.001$	2.07E+13	1371.86
1-1-3	Temperature (200 m)	3	BSS	0.60	6.72E+01	1 and 45	$p < 0.001$	1.36E+13	1380.66
1-1-4	Temperature (200 m)	0	NwS	0.25	1.57E+01	1 and 48	$p < 0.001$	2.57E+13	1497.09
1-1-5	Temperature (200 m)	0	FSC	0.25	1.63E+01	1 and 48	$p < 0.001$	2.54E+13	1496.62
1-1-6	Temperature (200 m)	0	IFR	0.01	2.99E-01	1 and 48	5.87E-01	3.38E+13	1510.90
1-1-7	Temperature (200 m)	3	BSO	0.57	6.04E+01	1 and 45	$p < 0.001$	1.44E+13	1383.56
1-1-8	Temperature (200 m)	1	NwSN	0.37	2.74E+01	1 and 47	$p < 0.001$	2.14E+13	1459.51
1-1-9	Temperature (200 m)	4	NwSS	0.41	3.04E+01	1 and 44	$p < 0.001$	1.99E+13	1370.03
1-1-10	Temperature (200 m)	6	RT	0.55	5.09E+01	1 and 42	$p < 0.001$	1.52E+13	1300.75
1-2-1	Salinity (200 m)	1	BS	0.66	9.01E+01	1 and 47	$p < 0.001$	1.16E+13	1429.55
1-2-2	Salinity (200 m)	1	BSN	0.54	5.43E+01	1 and 47	$p < 0.001$	1.58E+13	1444.38
1-2-3	Salinity (200 m)	1	BSS	0.63	7.85E+01	1 and 47	$p < 0.001$	1.27E+13	1433.88
1-2-4	Salinity (200 m)	5	NwS	0.61	6.81E+01	1 and 43	$p < 0.001$	1.30E+13	1322.19
1-2-5	Salinity (200 m)	6	FSC	0.39	2.63E+01	1 and 42	$p < 0.001$	2.06E+13	1314.31
1-2-6	Salinity (200 m)	0	IFR	0.05	2.74E+00	1 and 48	1.05E-01	3.22E+13	1508.44
1-2-7	Salinity (200 m)	3	BSO	0.67	9.06E+01	1 and 45	$p < 0.001$	1.12E+13	1371.75
1-2-8	Salinity (200 m)	7	NwSN	0.56	5.16E+01	1 and 41	$p < 0.001$	1.48E+13	1271.42
1-2-9	Salinity (200 m)	6	NwSS	0.65	7.73E+01	1 and 42	$p < 0.001$	1.18E+13	1289.75
1-2-10	Salinity (200 m)	6	RT	0.64	7.61E+01	1 and 42	$p < 0.001$	1.19E+13	1290.17
1-3-1	Sea Ice fraction (summer)	2	BS	0.10	5.36E+00	1 and 46	2.51E-02	3.04E+13	1447.53
1-3-2	Sea Ice fraction (summer)	2	BSN	0.10	5.35E+00	1 and 46	2.53E-02	3.04E+13	1447.54
1-3-3	Sea Ice fraction (summer)	4	BSS	0.11	5.39E+00	1 and 44	2.50E-02	3.00E+13	1388.91
1-3-4	Sea Ice fraction (summer)	1	NwS	0.04	2.10E+00	1 and 47	1.54E-01	3.25E+13	1479.86
1-4-1	Sea Ice fraction (winter)	2	BS	0.56	5.92E+01	1 and 46	$p < 0.001$	1.48E+13	1413.10
1-4-2	Sea Ice fraction (winter)	2	BSN	0.54	5.31E+01	1 and 46	$p < 0.001$	1.57E+13	1415.97
1-4-3	Sea Ice fraction (winter)	1	BSS	0.35	2.55E+01	1 and 47	$p < 0.001$	2.20E+13	1460.77
1-4-4	Sea Ice fraction (winter)	2	NwS	0.35	2.51E+01	1 and 46	$p < 0.001$	2.19E+13	1431.93
1-5-1	GPP	2	BS	0.45	3.69E+01	1 and 46	$p < 0.001$	1.88E+13	1424.56
1-5-2	GPP	3	BSN	0.40	3.02E+01	1 and 45	$p < 0.001$	2.02E+13	1399.46
1-5-3	GPP	2	BSS	0.36	2.62E+01	1 and 46	$p < 0.001$	2.16E+13	1431.18
1-5-4	GPP	5	NwS	0.00	9.20E-02	1 and 43	7.63E-01	3.35E+13	1364.83
1-5-5	GPP	6	FSC	0.08	3.87E+00	1 and 42	5.57E-02	3.07E+13	1331.79
1-5-6	GPP	5	IFR	0.06	2.88E+00	1 and 43	9.68E-02	3.15E+13	1362.01
1-5-7	GPP	8	BSO	0.21	1.09E+01	1 and 40	2.01E-03	2.63E+13	1267.09
1-5-8	GPP	5	NwSN	0.01	3.70E-01	1 and 43	5.46E-01	3.33E+13	1364.54
1-5-9	GPP	5	NwSS	0.00	3.43E-02	1 and 43	8.54E-01	3.35E+13	1364.89

Variables are taken from each station. Abbreviations of station and variable names are defined in S7 Table.

R^2: Coefficient of determination, DF: Degree of freedom of *F*-statistics, *RSS*: Residual sum of squares, AIC: Akaike's Information Criterion

S2 Table. Continued.

Model No.	Variable(s)		Station	R^2	*F*-statistics	DF	*p*-value	*RSS*	AIC
	x_1	Time lag							
1-6-1	GSP	2	BS	0.45	3.77E+01	1 and 46	$p < 0.001$	1.86E+13	1424.06
1-6-2	GSP	3	BSN	0.43	3.33E+01	1 and 45	$p < 0.001$	1.94E+13	1397.55
1-6-3	GSP	2	BSS	0.38	2.85E+01	1 and 46	$p < 0.001$	2.09E+13	1429.70
1-6-4	GSP	6	NwS	0.53	4.67E+01	1 and 42	$p < 0.001$	1.59E+13	1302.77
1-6-5	GSP	6	FSC	0.04	1.88E+00	1 and 42	1.78E-01	3.21E+13	1333.75
1-6-6	GSP	1	IFR	0.09	4.72E+00	1 and 47	3.50E-02	3.09E+13	1477.32
1-6-7	GSP	8	BSO	0.27	1.47E+01	1 and 40	$p < 0.001$	2.45E+13	1264.10
1-6-8	GSP	7	NwSN	0.55	5.09E+01	1 and 41	$p < 0.001$	1.50E+13	1271.76
1-6-9	GSP	9	NwSS	0.31	1.74E+01	1 and 39	$p < 0.001$	2.31E+13	1232.74
1-7	AMO index	6	AMO	0.51	4.28E+01	1 and 42	$p < 0.001$	1.66E+13	1304.74

Variables are taken from each station. Abbreviations of station and variable names are defined in S7 Table.

R^2: Coefficient of determination, DF: Degree of freedom of *F*-statistics, *RSS*: Residual sum of squares, AIC: Akaike's Information Criterion
