# Supplementary material for: Exploration of short-term predictions and long-term projections of Barents Sea cod biomass using statistical methods on data from dynamical models
Source: PLoS One. 2025 Jul 31;20(7):e0328762. doi: 10.1371/journal.pone.0328762 (PMC12312909; doi:10.1371/journal.pone.0328762)
Supplement: S3 Table — (PDF) [file pone.0328762.s003.pdf]

S3 Table. List of multiple regression models for total stock biomass of the NEA cod in the Barents Sea (TSB) and statistics of the regression models.

Model No.	Variable		Variable		interaction term	R^2	*F*-statistics	DF	*p*-value	*RSS*	AIC
	x_1	Time lag	x_2	Time lag							
2-1	Salinity (200 m)	1	Temperature (200m)	3	With	0.75	4.24E+01	3 and 43	$p < 0.001$	8.55E+12	1362.94
2-2	Salinity (200 m)	1	Temperature (200m)	3	Without	0.75	6.47E+01	2 and 44	$p < 0.001$	8.58E+12	1361.10
2-3	Salinity (200 m)	1	Sea Ice fraction (summer)	2	With	0.68	3.14E+01	3 and 44	$p < 0.001$	1.08E+13	1401.87
2-4	Salinity (200 m)	1	Sea Ice fraction (summer)	2	Without	0.68	4.79E+01	2 and 45	$p < 0.001$	1.08E+13	1400.05
2-5	Salinity (200 m)	1	Sea Ice fraction (winter)	2	With	0.72	3.72E+01	3 and 44	$p < 0.001$	9.58E+12	1396.17
2-6	Salinity (200 m)	1	Sea Ice fraction (winter)	2	Without	0.72	5.70E+01	2 and 45	$p < 0.001$	9.59E+12	1394.23
2-7	Temperature (200m)	3	GPP	2	With	0.69	3.17E+01	3 and 43	$p < 0.001$	1.05E+13	1372.74
2-8	Temperature (200m)	3	GPP	2	Without	0.68	4.74E+01	2 and 44	$p < 0.001$	1.07E+13	1371.57
2-9	Temperature (200m)	3	GSP	2	With	0.69	3.23E+01	3 and 43	$p < 0.001$	1.04E+13	1372.14
2-10	Temperature (200m)	3	GSP	2	Without	0.69	4.88E+01	2 and 44	$p < 0.001$	1.05E+13	1370.62

Variables are obtained in the Barents Sea Abbreviations of variable names are defined in S7 Table. R^2: Coefficient of determination, DF: Degree of freedom of *F*-statistics, *RSS*: Residual sum of squares, AIC: Akaike's Information Criterion
