# Supplementary material for: Exploration of short-term predictions and long-term projections of Barents Sea cod biomass using statistical methods on data from dynamical models
Source: PLoS One. 2025 Jul 31;20(7):e0328762. doi: 10.1371/journal.pone.0328762 (PMC12312909; doi:10.1371/journal.pone.0328762)
Supplement: S4 Table — (PDF) [file pone.0328762.s004.pdf]

S4 Table. Statistics of the regression coefficients of the simple regression models.

Model No.	variables	Regression coefficients		Standard error of the coefficients	*t*-value	*p*-value
1-1-1	Intercept	α_0	83981	7.56E+04	1.11E+00	2.73E-01
	x_1	α_1	1720546	1.89E+05	9.08E+00	$p < 0.001$
1-1-2	Intercept	α_0	85200	1.03E+05	8.31E-01	4.11E-01
	x_1	α_1	2184208	4.16E+05	5.25E+00	$p < 0.001$
1-1-3	Intercept	α_0	76914	8.06E+04	9.54E-01	3.45E-01
	x_1	α_1	1074885	1.31E+05	8.19E+00	$p < 0.001$
1-1-4	Intercept	α_0	4.48E-10	1.03E+05	4.33E-15	1.00E+00
	x_1	α_1	1526887	3.86E+05	3.96E+00	$p < 0.001$
1-1-5	Intercept	α_0	6.54E-10	1.03E+05	6.36E-15	1.00E+00
	x_1	α_1	848555	2.10E+05	4.03E+00	$p < 0.001$
1-1-6	Intercept	α_0	-6.01E-11	1.19E+05	-5.06E-16	1.00E+00
	x_1	α_1	170914	3.13E+05	5.46E-01	5.87E-01
1-1-7	Intercept	α_0	51776	8.28E+04	6.25E-01	5.35E-01
	x_1	α_1	1214140	1.56E+05	7.77E+00	$p < 0.001$
1-1-8	Intercept	α_0	24267	9.67E+04	2.51E-01	8.03E-01
	x_1	α_1	1239516	2.37E+05	5.23E+00	$p < 0.001$
1-1-9	Intercept	α_0	74832	1.00E+05	7.47E-01	4.59E-01
	x_1	α_1	1390836	2.52E+05	5.52E+00	$p < 0.001$
1-1-10	Intercept	α_0	-38037	9.06E+04	-4.20E-01	6.77E-01
	x_1	α_1	2205914	3.09E+05	7.13E+00	$p < 0.001$
1-2-1	Intercept	α_0	10102	7.11E+04	1.42E-01	8.88E-01
	x_1	α_1	15650787	1.65E+06	9.49E+00	$p < 0.001$
1-2-2	Intercept	α_0	12124	8.27E+04	1.47E-01	8.84E-01
	x_1	α_1	11925399	1.62E+06	7.37E+00	$p < 0.001$
1-2-3	Intercept	α_0	2900	7.43E+04	3.90E-02	9.69E-01
	x_1	α_1	15989173	1.80E+06	8.86E+00	$p < 0.001$

S4 Table. Continued.

Model No.	variables	Regression coefficients		Standard error of the coefficients	*t*-value	*p*-value
1-2-4	Intercept	α_0	178093	8.50E+04	2.09E+00	4.22E-02
	x_1	α_1	27452315	3.33E+06	8.26E+00	$p < 0.001$
1-2-5	Intercept	α_0	55636	1.07E+05	5.22E-01	6.04E-01
	x_1	α_1	11720659	2.29E+06	5.12E+00	$p < 0.001$
1-2-6	Intercept	α_0	-2.17E-08	1.16E+05	-1.87E-13	1.00E+00
	x_1	α_1	5860424	3.54E+06	1.65E+00	1.05E-01
1-2-7	Intercept	α_0	57256	7.31E+04	7.84E-01	4.37E-01
	x_1	α_1	15363600	1.61E+06	9.52E+00	$p < 0.001$
1-2-8	Intercept	α_0	230098	9.80E+04	2.35E+00	2.38E-02
	x_1	α_1	19447806	2.71E+06	7.18E+00	$p < 0.001$
1-2-9	Intercept	α_0	103088	8.10E+04	1.27E+00	2.10E-01
	x_1	α_1	15275892	1.74E+06	8.79E+00	$p < 0.001$
1-2-10	Intercept	α_0	17126	8.04E+04	2.13E-01	8.32E-01
	x_1	α_1	22734718	2.61E+06	8.73E+00	$p < 0.001$
1-3-1	Intercept	α_0	283016	1.70E+05	1.67E+00	1.02E-01
	x_1	α_1	-12538261	5.42E+06	-2.31E+00	2.51E-02
1-3-2	Intercept	α_0	282744	1.70E+05	1.67E+00	1.03E-01
	x_1	α_1	-5752809	2.49E+06	-2.31E+00	2.53E-02
1-3-3	Intercept	α_0	72336	1.26E+05	5.74E-01	5.69E-01
	x_1	α_1	-8821610490	3.80E+09	-2.32E+00	2.50E-02
1-3-4	Intercept	α_0	30659	1.21E+05	2.53E-01	8.02E-01
	x_1	α_1	-923419075	6.37E+08	-1.45E+00	1.54E-01
1-4-1	Intercept	α_0	2427024	3.26E+05	7.45E+00	$p < 0.001$
	x_1	α_1	-5502327	7.15E+05	-7.70E+00	$p < 0.001$
1-4-2	Intercept	α_0	2622407	3.70E+05	7.09E+00	$p < 0.001$
	x_1	α_1	-3789500	5.20E+05	-7.29E+00	$p < 0.001$
1-4-3	Intercept	α_0	1117693	2.43E+05	4.60E+00	$p < 0.001$
	x_1	α_1	-5039511	9.98E+05	-5.05E+00	$p < 0.001$
1-4-4	Intercept	α_0	783613	1.86E+05	4.22E+00	$p < 0.001$
	x_1	α_1	-28191119	5.63E+06	-5.01E+00	$p < 0.001$

S4 Table. Continued.

Model No.	variables	Regression coefficients		Standard error of the coefficients	*t*-value	*p*-value
1-5-1	Intercept	α_0	20381	9.24E+04	2.21E-01	8.26E-01
	x_1	α_1	29429	4.85E+03	6.07E+00	$p < 0.001$
1-5-2	Intercept	α_0	37306	9.80E+04	3.81E-01	7.05E-01
	x_1	α_1	20121	3.66E+03	5.49E+00	$p < 0.001$
1-5-3	Intercept	α_0	12249	9.89E+04	1.24E-01	9.02E-01
	x_1	α_1	37554	7.34E+03	5.12E+00	$p < 0.001$
1-5-4	Intercept	α_0	-11796	1.32E+05	-8.96E-02	9.29E-01
	x_1	α_1	3053	1.01E+04	3.03E-01	7.63E-01
1-5-5	Intercept	α_0	-31692	1.29E+05	-2.45E-01	8.07E-01
	x_1	α_1	14539	7.39E+03	1.97E+00	5.57E-02
1-5-6	Intercept	α_0	210	1.28E+05	1.64E-03	9.99E-01
	x_1	α_1	13699	8.07E+03	1.70E+00	9.68E-02
1-5-7	Intercept	α_0	15103	1.26E+05	1.20E-01	9.05E-01
	x_1	α_1	32574	9.86E+03	3.30E+00	2.01E-03
1-5-8	Intercept	α_0	-12812	1.31E+05	-9.76E-02	9.23E-01
	x_1	α_1	4837	7.95E+03	6.08E-01	5.46E-01
1-5-9	Intercept	α_0	-10617	1.32E+05	-8.06E-02	9.36E-01
	x_1	α_1	-1822	9.84E+03	-1.85E-01	8.54E-01
1-6-1	Intercept	α_0	21804	9.19E+04	2.37E-01	8.14E-01
	x_1	α_1	54726	8.91E+03	6.14E+00	$p < 0.001$
1-6-2	Intercept	α_0	36633	9.60E+04	3.82E-01	7.05E-01
	x_1	α_1	46600	8.08E+03	5.77E+00	$p < 0.001$
1-6-3	Intercept	α_0	18359	9.75E+04	1.88E-01	8.51E-01
	x_1	α_1	52651	9.87E+03	5.34E+00	$p < 0.001$
1-6-4	Intercept	α_0	101236	9.42E+04	1.07E+00	2.89E-01
	x_1	α_1	42009	6.15E+03	6.84E+00	$p < 0.001$
1-6-5	Intercept	α_0	-30489	1.32E+05	-2.31E-01	8.19E-01
	x_1	α_1	14930	1.09E+04	1.37E+00	1.78E-01

S4 Table. Continued.

Model No.	variables	Regression coefficients		Standard error of the coefficients	t-value	p-value
1-6-6	Intercept	α_0	-5114	1.16E+05	-4.42E-02	9.65E-01
	x_1	α_1	21622	9.96E+03	2.17E+00	3.50E-02
1-6-7	Intercept	α_0	31656	1.22E+05	2.61E-01	7.96E-01
	x_1	α_1	31231	8.15E+03	3.83E+00	$p < 0.001$
1-6-8	Intercept	α_0	94463	9.34E+04	1.01E+00	3.18E-01
	x_1	α_1	25019	3.51E+03	7.13E+00	$p < 0.001$
1-6-9	Intercept	α_0	21795	1.21E+05	1.81E-01	8.57E-01
	x_1	α_1	44032	1.06E+04	4.17E+00	$p < 0.001$
1-7	Intercept	α_0	42273	9.52E+04	4.44E-01	6.59E-01
	x_1	α_1	2426374	3.71E+05	6.54E+00	$p < 0.001$
