# Supplementary material for: Exploration of short-term predictions and long-term projections of Barents Sea cod biomass using statistical methods on data from dynamical models
Source: PLoS One. 2025 Jul 31;20(7):e0328762. doi: 10.1371/journal.pone.0328762 (PMC12312909; doi:10.1371/journal.pone.0328762)
Supplement: S5 Table — (PDF) [file pone.0328762.s005.pdf]

S5 Table. Statistics of the regression coefficients of the multiple regression models.

Model No.	variables	Regression coefficients		Standard error of the coefficients	*t*-value	*p*-value	*VIF*
2-1	Intercept	α_0	-2694	9.29E+04	-2.90E-02	9.77E-01	-
	x_1	α_1	11021321	2.67E+06	4.13E+00	$p < 0.001$	2.94
	x_2	α_2	754776	2.96E+05	2.55E+00	1.44E-02	3.26
	x_1*x_2	α_3	1666756	4.44E+06	3.76E-01	7.09E-01	1.21
2-2	Intercept	α_0	21400	6.66E+04	3.22E-01	7.49E-01	-
	x_1	α_1	10907264	2.63E+06	4.15E+00	$p < 0.001$	2.90
	x_2	α_2	791778	2.76E+05	2.86E+00	6.39E-03	2.90
2-3	Intercept	α_0	65047	1.07E+05	6.10E-01	5.45E-01	-
	x_1	α_1	16407395	2.41E+06	6.81E+00	$p < 0.001$	2.06
	x_2	α_2	-3596418	3.76E+06	-9.58E-01	3.43E-01	1.29
	x_1*x_2	α_3	-37576240	9.30E+07	-4.04E-01	6.88E-01	2.40
2-4	Intercept	α_0	62482	1.05E+05	5.93E-01	5.56E-01	-
	x_1	α_1	15744953	1.75E+06	9.01E+00	$p < 0.001$	1.10
	x_2	α_2	-3015670	3.44E+06	-8.77E-01	3.85E-01	1.10
2-5	Intercept	α_0	960488	4.18E+05	2.30E+00	2.65E-02	-
	x_1	α_1	13392696	7.39E+06	1.81E+00	7.67E-02	21.82
	x_2	α_2	-2221273	9.00E+05	-2.47E+00	1.75E-02	2.34
	x_1*x_2	α_3	-4153517	1.68E+07	-2.47E-01	8.06E-01	18.82
2-6	Intercept	α_0	993124	3.93E+05	2.53E+00	1.50E-02	-
	x_1	α_1	11667282	2.36E+06	4.95E+00	$p < 0.001$	2.27
	x_2	α_2	-2260970	8.76E+05	-2.58E+00	1.32E-02	2.27
2-7	Intercept	α_0	124404	9.34E+04	1.33E+00	1.90E-01	-
	x_1	α_1	1444705	2.52E+05	5.73E+00	$p < 0.001$	1.86
	x_2	α_2	9879	5.12E+03	1.93E+00	6.05E-02	1.74
	x_1*x_2	α_3	-9811	1.12E+04	-8.78E-01	3.85E-01	1.10
2-8	Intercept	α_0	73127	7.26E+04	1.01E+00	3.20E-01	-
	x_1	α_1	1374632	2.39E+05	5.76E+00	$p < 0.001$	1.73
	x_2	α_2	11035	4.94E+03	2.23E+00	3.06E-02	1.73

VIF: Variance inflation factor

S5 Table. Continued.

Model No.	variables	Regression coefficients		Standard error of the coefficients	*t*-value	*p*-value	*VIF*
2-9	Intercept	α_0	110368	9.21E+04	1.20E+00	2.37E-01	-
	x_1	α_1	1403268	2.45E+05	5.73E+00	$p < 0.001$	2.07
	x_2	α_2	20355	9.29E+03	2.19E+00	3.40E-02	1.92
	$x_1 * x_2$	α_3	-13780	2.07E+04	-6.66E-01	5.09E-01	1.11
2-10	Intercept	α_0	72410	7.19E+04	1.01E+00	3.19E-01	-
	x_1	α_1	1356628	2.33E+05	5.82E+00	$p < 0.001$	1.91
	x_2	α_2	21895	8.94E+03	2.45E+00	1.84E-02	1.91

VIF: Variance inflation factor
