# Supplementary material for: Exploration of short-term predictions and long-term projections of Barents Sea cod biomass using statistical methods on data from dynamical models
Source: PLoS One. 2025 Jul 31;20(7):e0328762. doi: 10.1371/journal.pone.0328762 (PMC12312909; doi:10.1371/journal.pone.0328762)
Supplement: S6 Table — (PDF) [file pone.0328762.s006.pdf]

S6 Table. Correlations of variables used in the multiple regression models.

Model No. 2-1	TSB	Salinity (200m)	Temperature (200m)	Salinity (200m) × Temperature (200m)
TSB	-	-	-	-
Salinity (200m)	0.84 ($p < 0.001$)	-	-	-
Temperature (200m)	0.80 ($p < 0.001$)	0.81 ($p < 0.001$)	-	-
Salinity (200m) × Temperature (200m)	0.32 (p = 2.97E-02)	0.26 (p = 7.19E-02)	0.40 (p = 5.00E-03)	-

Model No. 2-2	TSB	Salinity (200m)	Temperature (200m)
TSB	-	-	-
Salinity (200m)	0.84 ($p < 0.001$)	-	-
Temperature (200m)	0.80 ($p < 0.001$)	0.81 ($p < 0.001$)	-

Model No. 2-3	TSB	Salinity (200m)	Sea Ice fraction (summer)	Salinity (200m) × Sea ice fraction (summer)
TSB	-	-	-	-
Salinity (200m)	0.82 ($p < 0.001$)	-	-	-
Sea Ice fraction (summer)	-0.32 (p = 2.51E-02)	-0.31 (p = 3.35E-02)	-	-
Salinity (200m) × Sea ice fraction (summer)	0.59 ($p < 0.001$)	0.72 ($p < 0.001$)	-0.47 ($p < 0.001$)	-

Model No. 2-4	TSB	Salinity (200m)	Sea Ice fraction (summer)
TSB	-	-	-
Salinity (200m)	0.82 ($p < 0.001$)	-	-
Sea Ice fraction (summer)	-0.32 (p = 2.51E-02)	-0.31 (p = 3.35E-02)	-

Model No. 2-5	TSB	Salinity (200m)	Sea Ice fraction (winter)	Salinity (200m) × Sea Ice fraction (winter)
TSB	-	-	-	-
Salinity (200m)	0.82 ($p < 0.001$)	-	-	-
Sea Ice fraction (winter)	-0.75 ($p < 0.001$)	-0.75 ($p < 0.001$)	-	-
Salinity (200m) × Sea Ice fraction (winter)	0.79 ($p < 0.001$)	0.97 ($p < 0.001$)	-0.70 ($p < 0.001$)	-

Abbreviations of variable names are defined in S7 Table.

S6 Table. Continued.

Model No. 2-6	TSB	Salinity (200m)	Sea Ice fraction (winter)
TSB	-	-	-
Salinity (200m)	0.82 ($p < 0.001$)	-	-
Sea Ice fraction (winter)	-0.75 ($p < 0.001$)	-0.75 ($p < 0.001$)	-

Model No. 2-7	TSB	Temperature (200m)	GPP	Temperature (200m) × GPP
TSB	-	-	-	-
Temperature (200m)	0.78 ($p < 0.001$)	-	-	-
GPP	0.66 ($p < 0.001$)	0.65 ($p < 0.001$)	-	-
Temperature (200m) × GPP	0.11 (p = 4.55E-01)	0.29 (p = 4.76E-02)	0.14 (p = 3.47E-01)	-

Model No. 2-8	TSB	Temperature (200m)	GPP
TSB	-	-	-
Temperature (200m)	0.78 ($p < 0.001$)	-	-
GPP	0.66 ($p < 0.001$)	0.65 ($p < 0.001$)	-

Model No. 2-9	TSB	Temperature (200m)	GSP	Temperature (200m) × GSP
TSB	-	-	-	-
Temperature (200m)	0.78 ($p < 0.001$)	-	-	-
GSP	0.66 ($p < 0.001$)	0.69 ($p < 0.001$)	-	-
Temperature (200m) × GSP	0.10 (p = 4.92E-01)	0.31 (p = 3.29E-02)	0.16 (p = 2.76E-01)	-

Model No. 2-10	TSB	Temperature (200m)	GSP
TSB	-	-	-
Temperature (200m)	0.78 ($p < 0.001$)	-	-
GSP	0.66 ($p < 0.001$)	0.69 ($p < 0.001$)	-

Abbreviations of variable names are defined in S7 Table.
