# Supplementary figures and images for: Exploration of short-term predictions and long-term projections of Barents Sea cod biomass using statistical methods on data from dynamical models
Source: PLoS One. 2025 Jul 31;20(7):e0328762. doi: 10.1371/journal.pone.0328762 (PMC12312909; doi:10.1371/journal.pone.0328762)

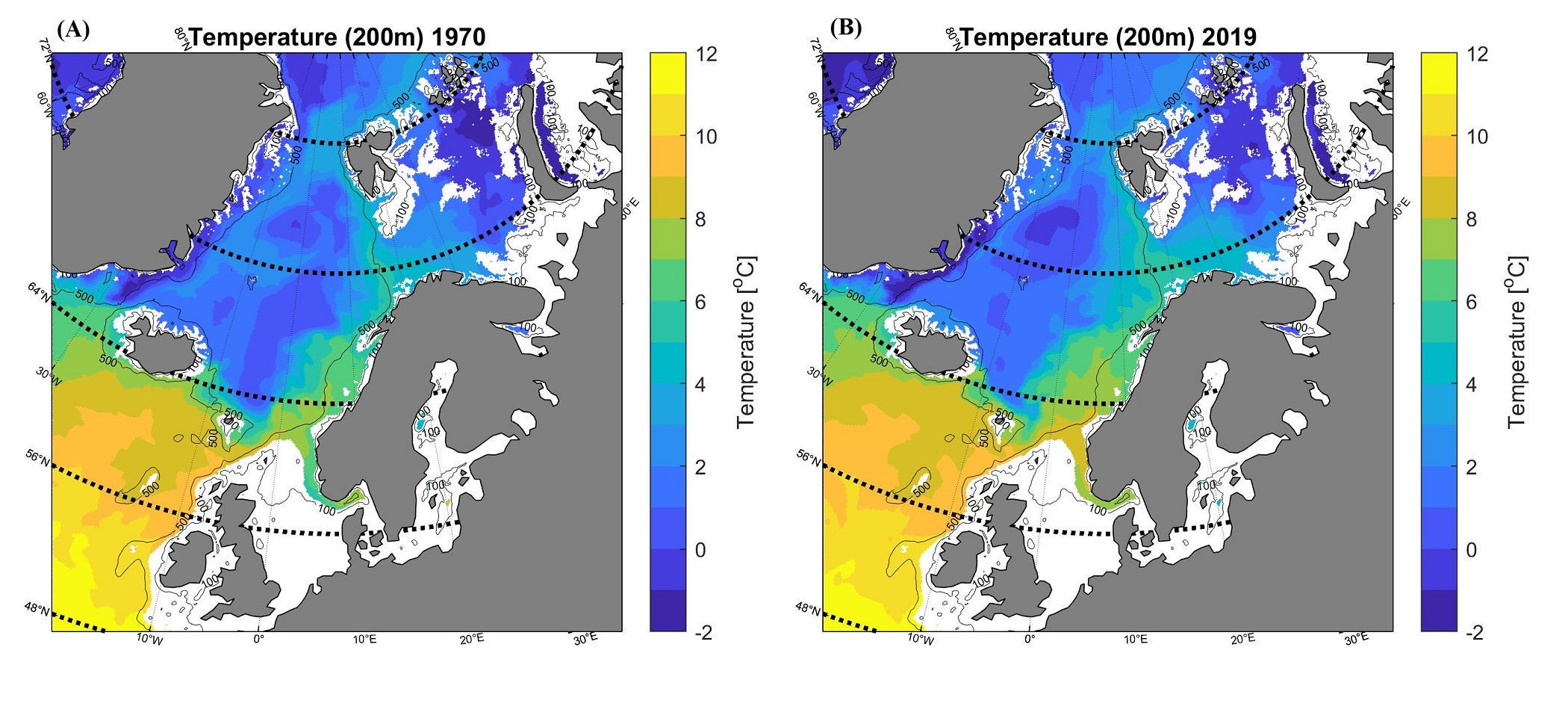

Supplement: S1 Fig — Two representative maps (A) in 1970, and (B) in 2019 are given. Data is also available between 1971–2018. (TIF) [file pone.0328762.s007.tif]

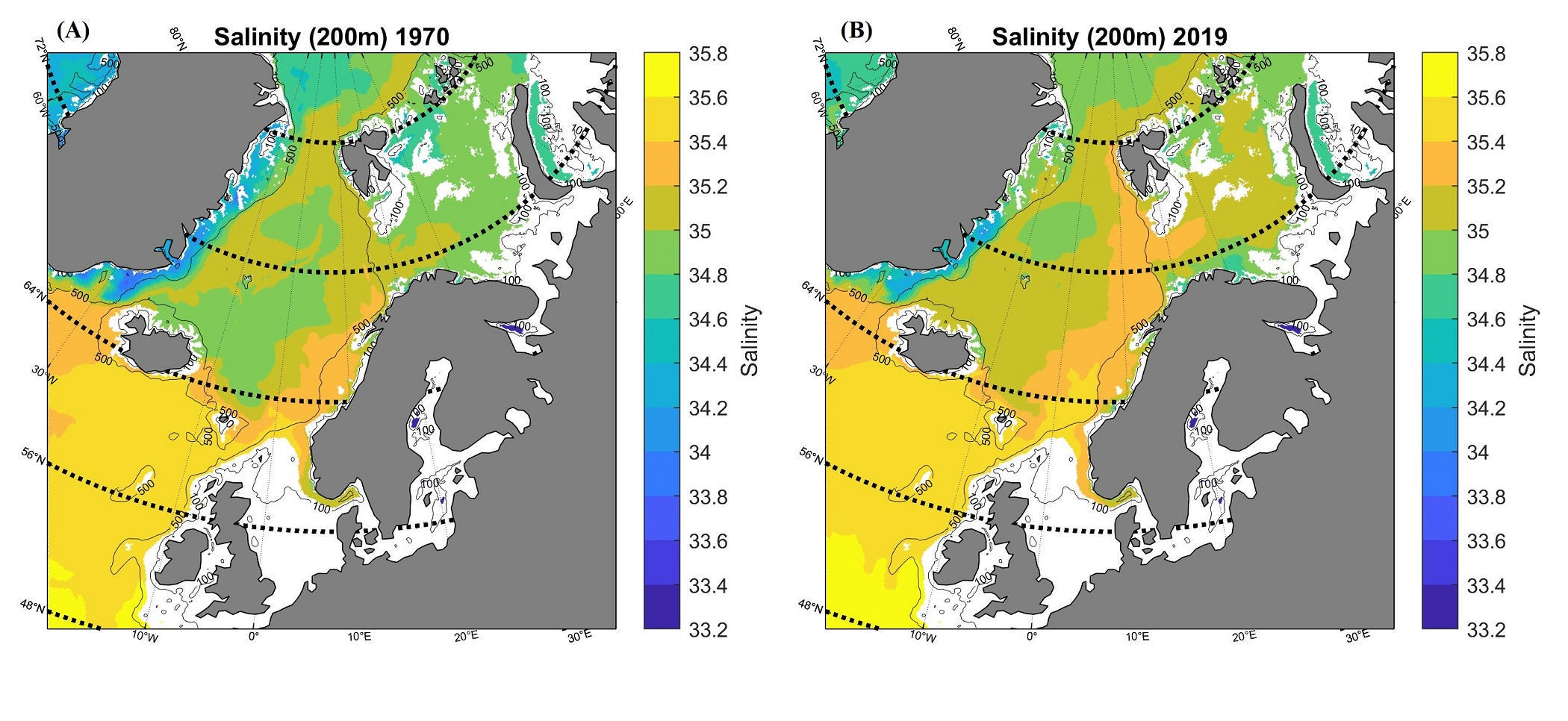

Supplement: S2 Fig — Two representative maps (A) in 1970, and (B) in 2019 are given. Data is also available between 1971–2018. (TIF) [file pone.0328762.s008.tif]

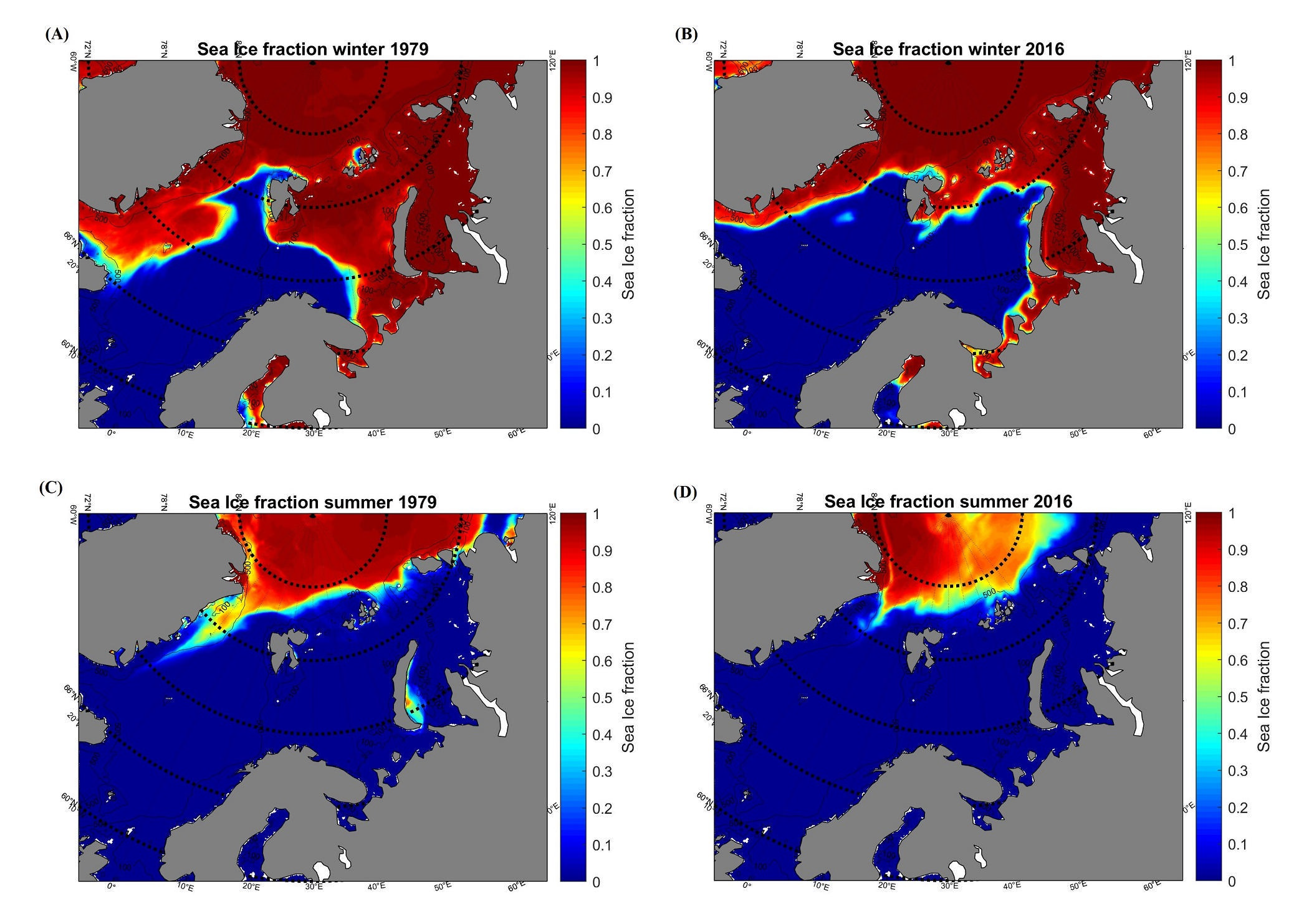

Supplement: S3 Fig — Representative maps are given; (A) maximum ice cover in 1979 in winter, and (B) minimum ice cover in 2016 in winter during recent years, (C) ice cover in 1979 in summer, (D) ice cover in 2016 in summer. Data is also available between 1970–2019. (TIF) [file pone.0328762.s009.tif]

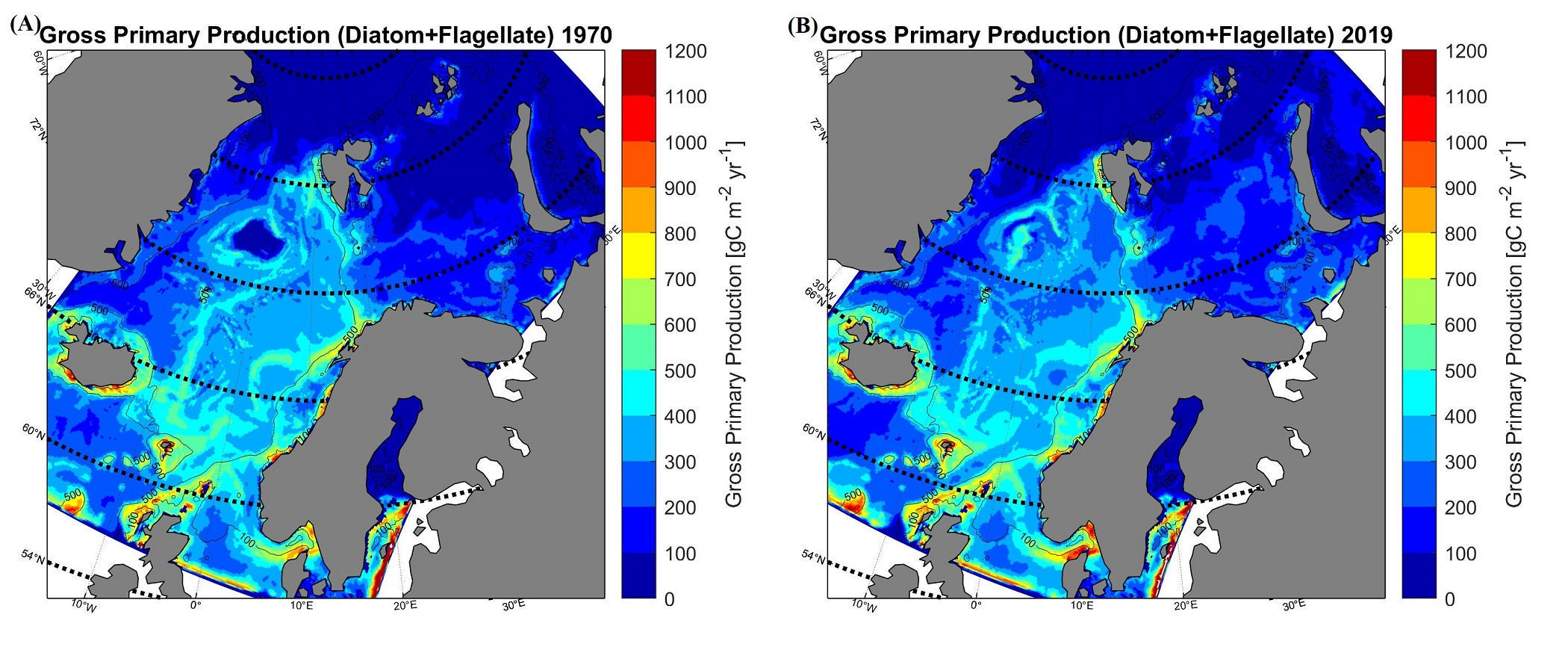

Supplement: S4 Fig — Two representative maps (A) in 1970, and (B) in 2019 are given. Data is also available between 1971–2018. (TIF) [file pone.0328762.s010.tif]

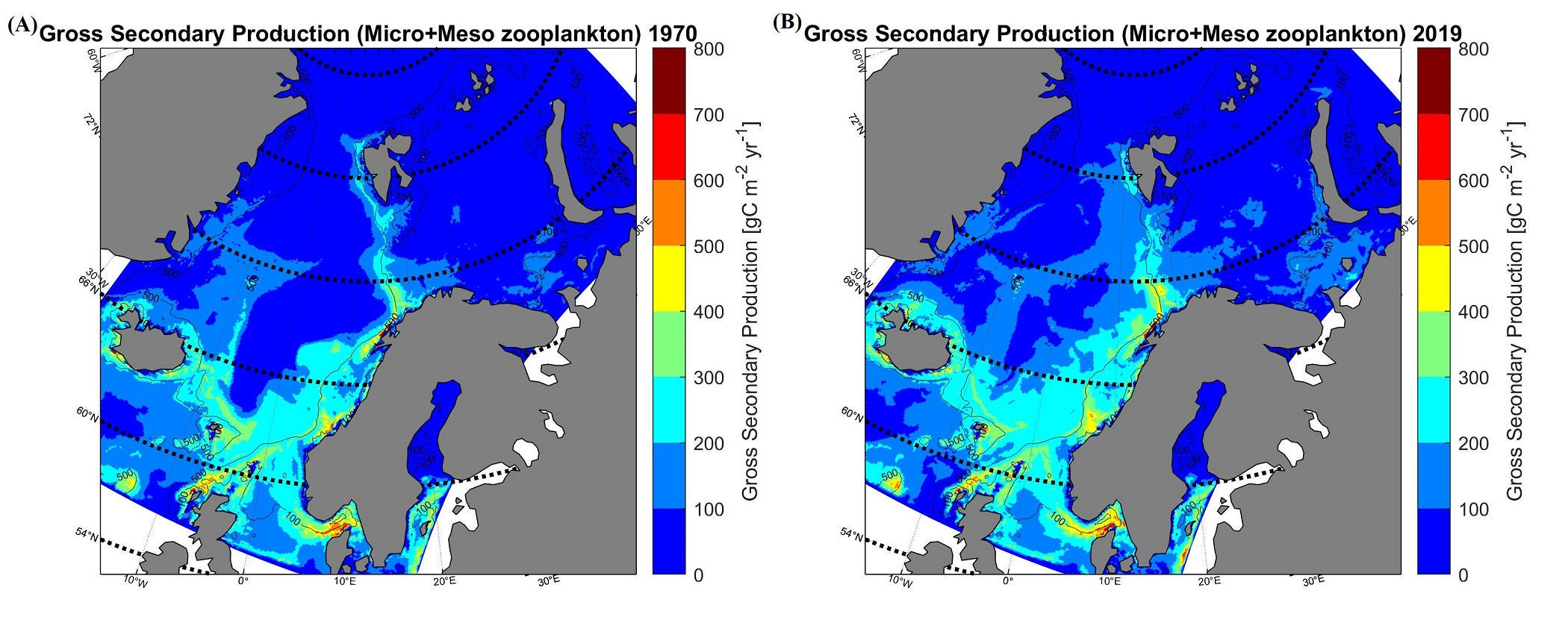

Supplement: S5 Fig — Two representative maps (A) in 1970, and (B) in 2019 are given. Data is also available between 1971–2018. (TIF) [file pone.0328762.s011.tif]

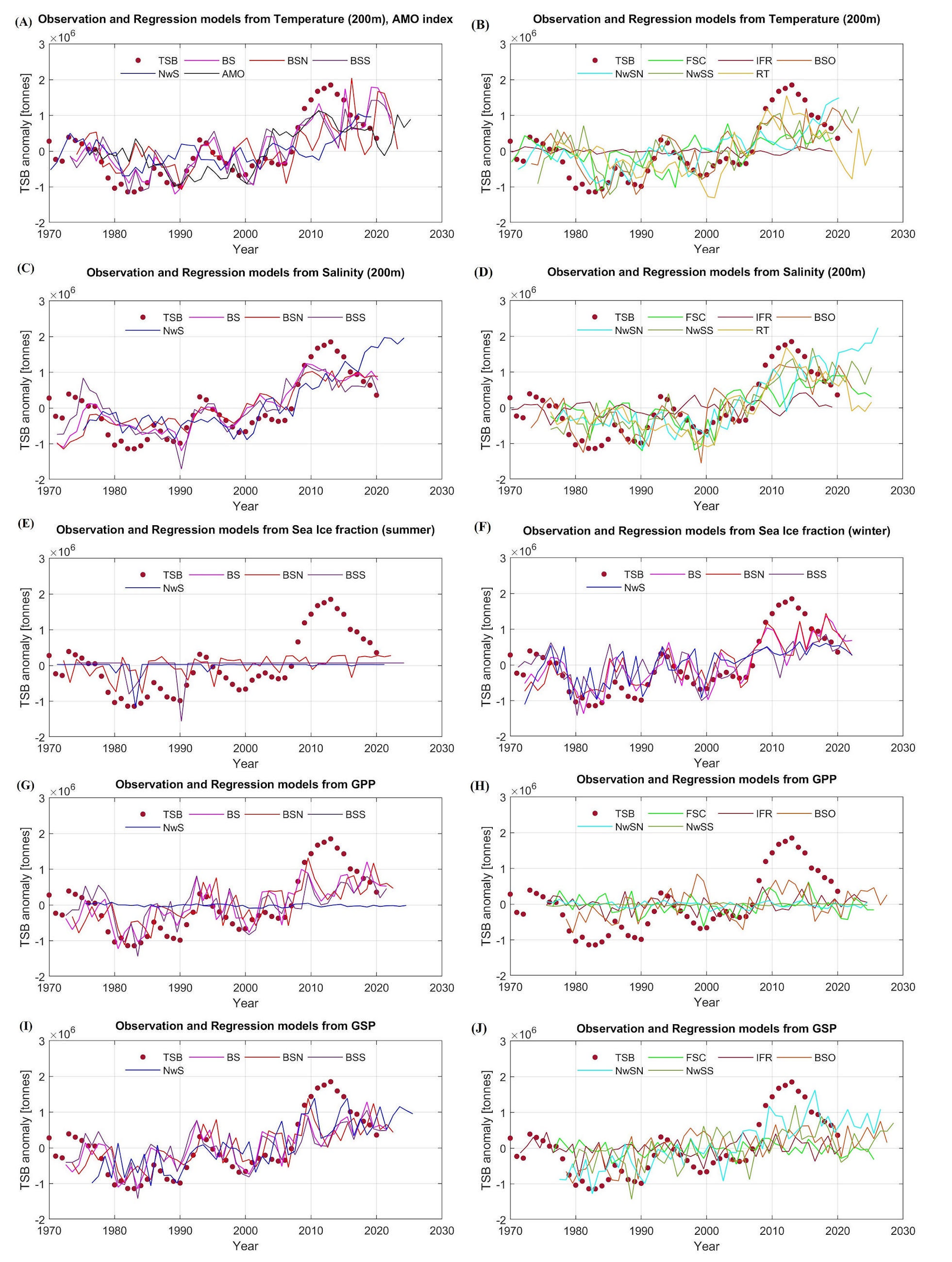

Supplement: S6 Fig — In each figure, spots show the total stock biomass of the NEA cod (TSB), and solid line shows TSB estimated by regression models. Each regression model is constructed by variables below: (A) temperature at 200m depth in the Barents Sea (BS) and the Norwegian Sea (NwS), and AMO index (B) temperature along the NAC/NwAC, (C) salinity at 200m depth at BS and NwS, (D) salinity along the NAC/NwAC, (E) sea ice fraction in summer at BS and NwS, (F) sea ice fraction in winter at BS and NwS, (G) GPP at BS and NwS (H) GPP along the NAC/NwAC, (I) GSP at BS and NwS, and (J) GSP along the NAC/NwAC. Anomalies are relative to 1970–2019. Abbreviations of focus area and variable names are defined in Fig 1. (TIF) [file pone.0328762.s012.tif]

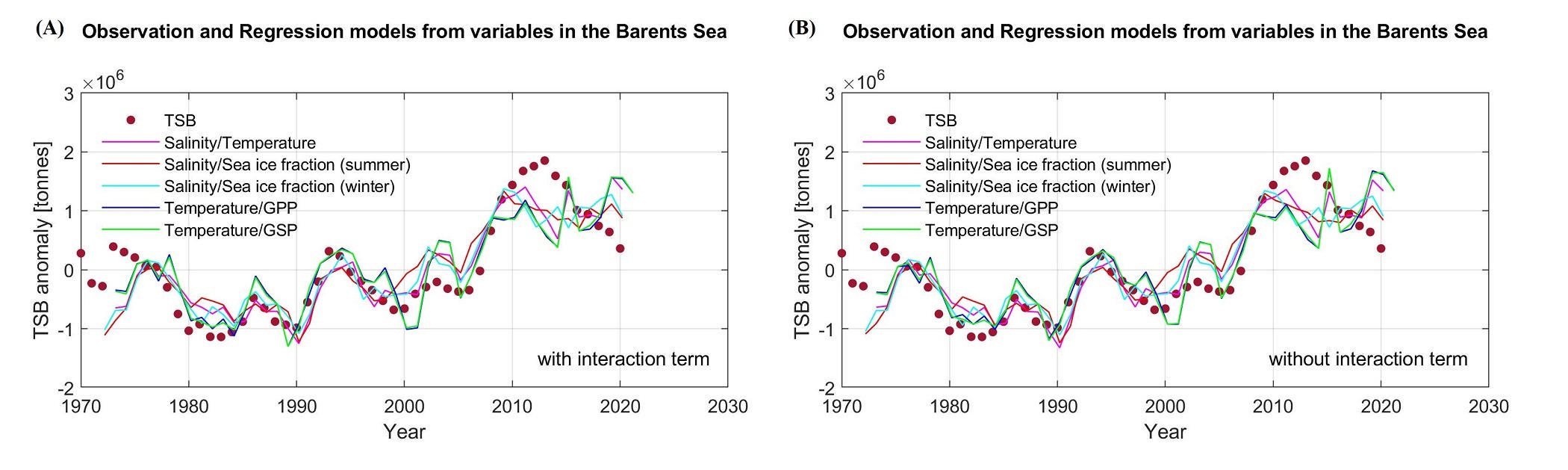

Supplement: S7 Fig — In each figure, spots show the total stock biomass of the NEA cod (TSB), and solid line shows TSB estimated by multiple regression models. Each regression model is constructed by two variables, which are obtained in the Barents Sea. There are two types of multiple regression models: (A) one includes an interaction term, and (B) another has no interaction term. Anomalies are relative to 1970–2019. (TIF) [file pone.0328762.s013.tif]

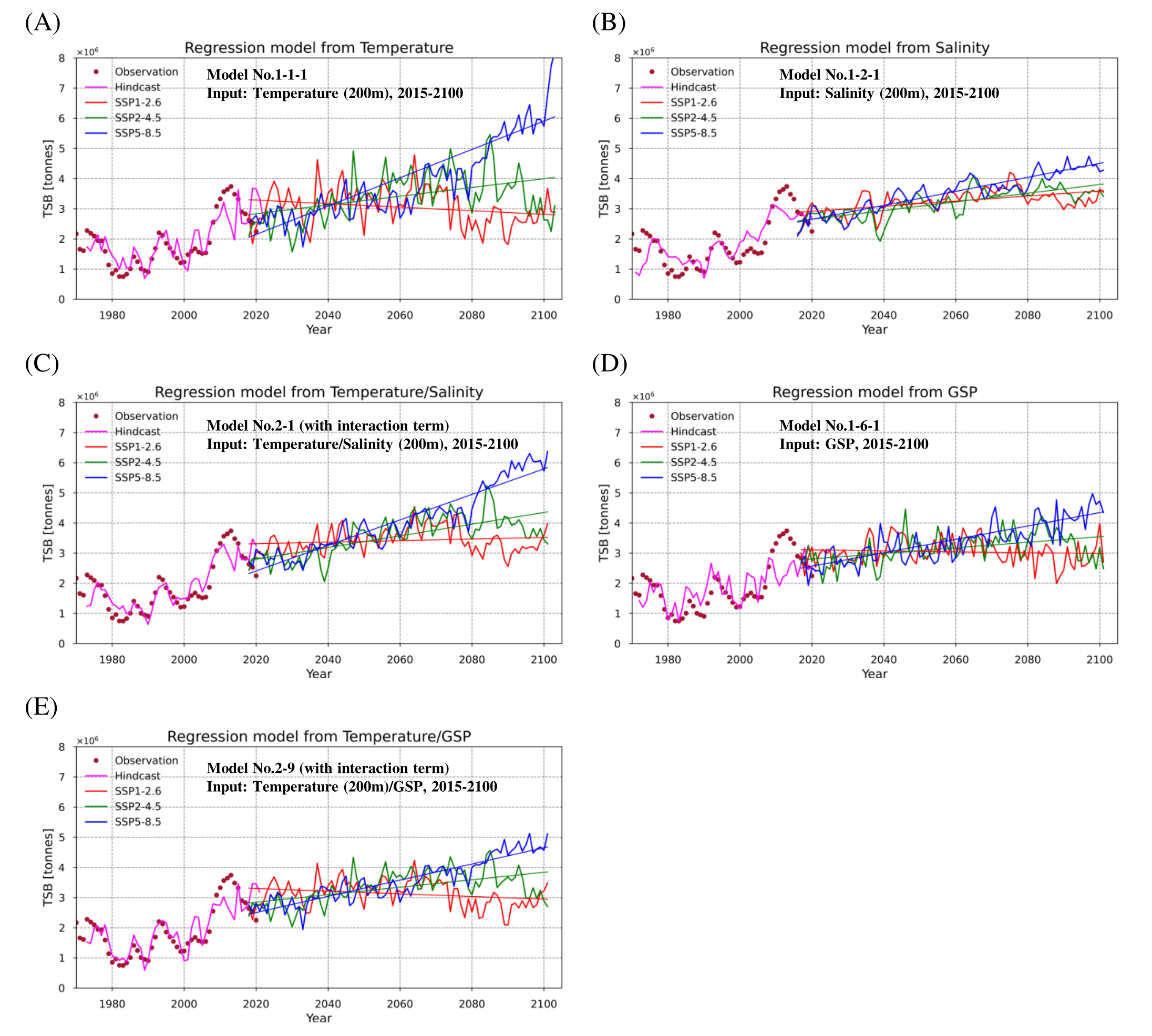

Supplement: S8 Fig — TSB estimated with regression models using variables with future climate scenarios: SSP1–2.6 (red lines), SSP2–4.5 (green lines), SSP5–8.5 (blue lines), observations (spots) and TSB predicted from hindcast (pink lines) are shown. TSB is calculated from (A) temperature with regression model No.1-1-1, (B) salinity with regression model No. 1-2-1, (C) temperature and salinity with regression model No. 2–1 (with an interaction term), (D) gross secondary production with regression model No. 1-6-1, (E) temperature and gross secondary production with regression model No. 2–9 (with an interaction term). Note that bias correction for temperature, salinity, GSP in projections is employed (see “Application of regression models to downscaled climate projections” in the “Discussion” for more details on bias correction). (TIF) [file pone.0328762.s014.tif]

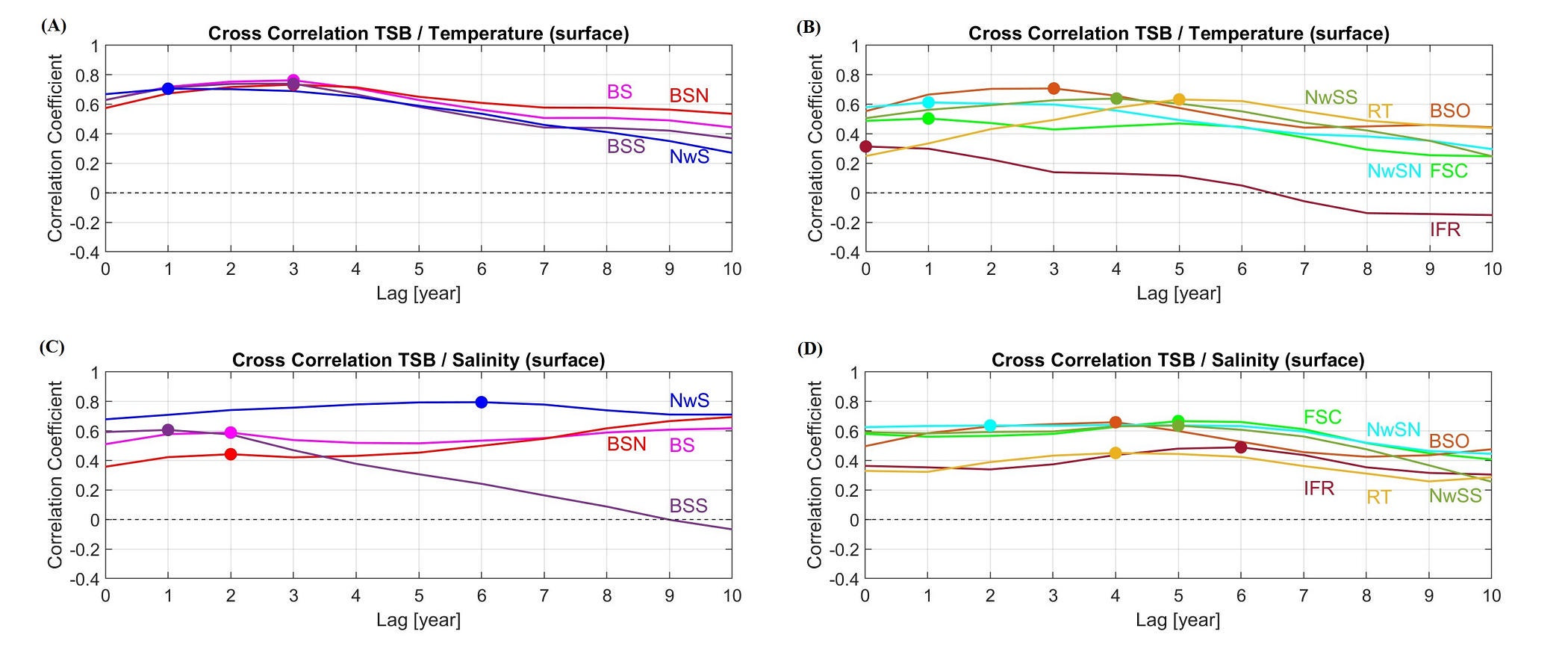

Supplement: S9 Fig — Cross correlations between TSB and (A) temperature at surface in the Barents Sea (BS) and the Norwegian Sea (NwS), (B) temperature along the NAC/NwAC, (C) salinity at surface in the BS and NwS, (D) salinity along the NAC/NwAC, are shown. The spots show the maximum correlation. Abbreviations of focus area names are defined in Fig 1. (TIF) [file pone.0328762.s015.tif]

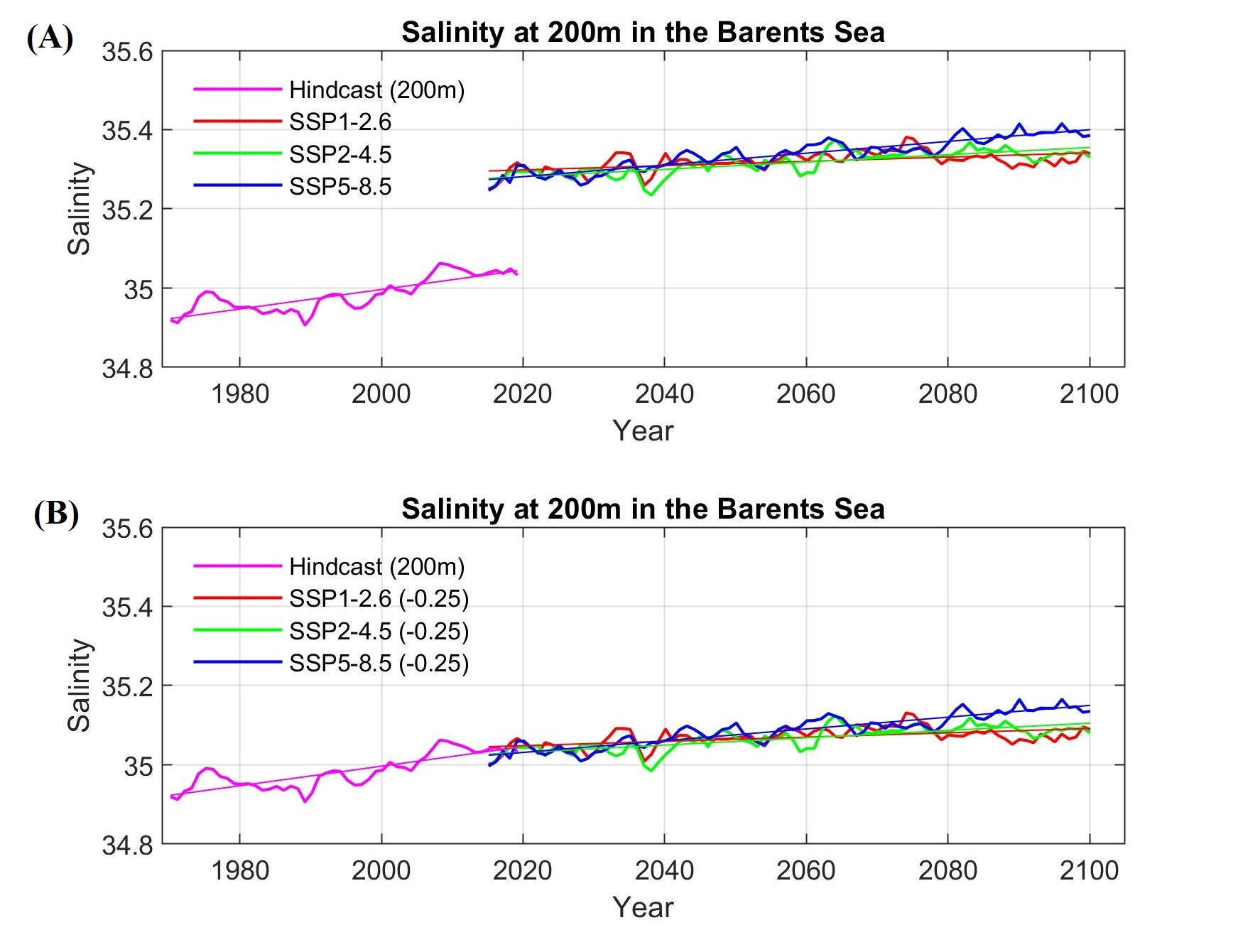

Supplement: S10 Fig — (A) Time series of salinity at 200 m depth in the Barents Sea (original data). Salinity from hindcast simulation (pink line), and from projections (red, green, blue) are shown. (B) A bias correction for salinity in projections is employed (salinity – 0.25 psu). (TIF) [file pone.0328762.s016.tif]

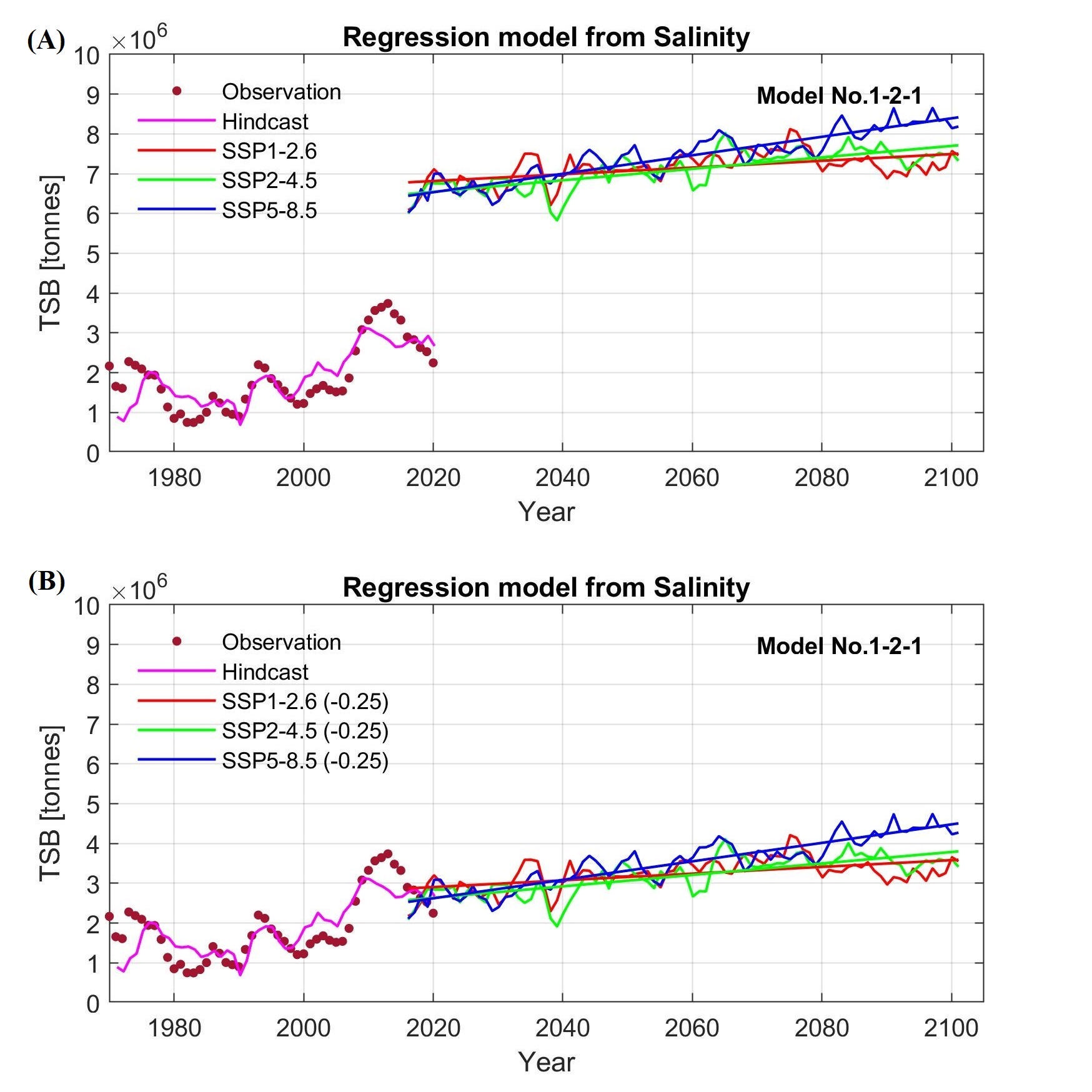

Supplement: S11 Fig — (A) Comparison of TSB estimated from regression model No. 1-2-1, based on salinity. Observation (spots), TSB predicted from hindcast simulation (pink line), and TSB projected from future climate scenarios (red, green, blue lines) are shown. (B) A bias correction for salinity in projections is employed (salinity – 0.25 psu). (TIF) [file pone.0328762.s017.tif]
