# Supplementary material for: Exploration of short-term predictions and long-term projections of Barents Sea cod biomass using statistical methods on data from dynamical models
Source: PLoS One. 2025 Jul 31;20(7):e0328762. doi: 10.1371/journal.pone.0328762 (PMC12312909; doi:10.1371/journal.pone.0328762)
Supplement: S1 File — (PDF) [file pone.0328762.s018.pdf]

S1 File. Supplementary Text.

The equations which are used in the Materials and Methods section are presented.

1 Regression Analysis

To estimate TSB of the NEA cod, simple/multiple linear regression models are constructed with different variables, obtained from ocean model/ecosystem model. The equation of the linear regression model is given as

$$y_a = \alpha_0 + \alpha_1 x_{1,a-b} + \alpha_2 x_{2,a-b} + \cdots + \alpha_k x_{k,a-b} + \varepsilon$$

where y_a is the response variable/predictand (TSB; year = a), α_0 is the y intercept, α_k is the regression coefficient, $x_{k,a-b}$ is the explanatory variable/predictor (e.g., temperature, salinity, GPP etc.) which can predict predictand by b year(s), k is the number of explanatory variables, and ε is the residual. In other words, the equation of a simple linear regression model is given as

$$y_a = \alpha_0 + \alpha_1 x_{1,a-b} + \varepsilon,$$

and the equation of a multiple linear regression model with two explanatory variables/predictors is given as

$$y_a = \alpha_0 + \alpha_1 x_{1,a-b} + \alpha_2 x_{2,a-b} + \varepsilon.$$

Moreover, the equation of a multiple linear regression model, which has an interaction term, is given as

$$y_a = \alpha_0 + \alpha_1 x_{1,a-b} + \alpha_2 x_{2,a-b} + \alpha_3 x_{1,a-b} x_{2,a-b} + \varepsilon.$$

2 Statistics of the linear regression models

To evaluate the linear regression models, statistics of the regression models, such as coefficient of determination (R^2), F-statistics, p-value, residual sum of squares (RSS), Akaike Information Criterion (AIC) and delta AIC are calculated. Moreover, statistics of the regression coefficients are also calculated to evaluate the regression coefficients of the models. These equations are based on [1-4]. For simplicity, the equations used for the simple regression models (k = 1) are presented here.

The equation of a simple linear regression model:

$$y_i = \alpha_0 + \alpha_1 x_i + \varepsilon_i \text{ for } i = 1, 2, \ldots, n \ (n\text{: sample size})$$

Mean of x:

$$\bar{x} = \frac{1}{n}\sum_{i=1}^{n} x_i$$

Mean of y:

$$\bar{y} = \frac{1}{n}\sum_{i=1}^{n} y_i$$

Sum of squares of x:

$$SS_x = \sum_{i=1}^{n} (x_i - \bar{x})^2$$

Sum of squares of y:

$$SS_y = \sum_{i=1}^{n} (y_i - \bar{y})^2$$

Sum of cross products:

$$S_{xy} = \sum_{i=1}^{n} (x_i - \bar{x})(y_i - \bar{y})$$

The estimate of α_1 (slope):

$$\alpha_1 = \frac{S_{xy}}{SS_x}$$

The estimate of α_0 (y intercept):

$$\alpha_0 = \bar{y} - \alpha_1 \bar{x}$$

The estimate of y:

$$\hat{y}_i = \alpha_0 + \alpha_1 x_i$$

The estimated residual:

$$\varepsilon_i = y_i - \hat{y}_i = y_i - \alpha_0 - \alpha_1 x_i$$

The residual sum of squares (*RSS*)/Error sum of squares (*SSE*):

$$RSS(SSE) = \sum_{i=1}^{n} {\varepsilon_i}^2 = \sum_{i=1}^{n} (y_i - \hat{y}_i)^2 = \sum_{i=1}^{n} (y_i - \alpha_0 - \alpha_1 x_i)^2$$

Mean square error (*MSE*):

$$MSE = \frac{RSS}{n - 2}$$

Standard error of the regression/standard deviation of residuals:

$$s = \sqrt{MSE}$$

The standard error of α_1 (slope):

$$SE(\alpha_1) = \frac{s}{\sqrt{SS_x}}$$

The standard error of α_0 (y intercept):

$$SE(\alpha_0) = s\sqrt{\frac{1}{n} + \frac{\bar{x}^2}{SS_x}}$$

The t-value of α_0 (y intercept):

$$t_0 = \frac{\alpha_0}{SE(\alpha_0)}$$

The t-value of α_1 (slope):

$$t_1 = \frac{\alpha_1}{SE(\alpha_1)}$$

The t-values follow a t-distribution with n - m degrees of freedom (DF), and m ($m = k + 1$) is the number of parameters including the y intercept in the model (e.g., $m = 2$ for the simple linear regression model). For the simple regression modes, m will be 2, therefore, t-values follow a t-distribution with DF = n - 2. The t-values can be used to assess the significance of the regression coefficient. In general, the regression coefficient will be considered to be significant when $|t| > 2$, and to be nonsignificant when $|t| < 2$ [4].

The Pearson correlation coefficient between x and y:

$$r = \frac{S_{xy}}{\sqrt{SS_x SS_y}}$$

Coefficient of determination:

$$R^2 = \frac{{S_{xy}}^2}{SS_x SS_y} = r^2$$

R^2 is a number between 0 and 1, and it indicates what percentage of the variation in the response variable y can be explained by the explanatory variable(s) x in the regression model [2,4].

F-statistics:

$$F = \frac{(SS_y - RSS)/(m-1)}{RSS/(n-m)}$$

follows an F distribution with DF = (m - 1, n - m), and m (m = k + 1) is the number of parameters in the model. For the simple regression modes, m will be 2, therefore,

$$F = \frac{(SS_y - RSS)/(2-1)}{RSS/(n-2)} = \frac{(SS_y - RSS)/1}{RSS/(n-2)}$$

and F-statistics follows an F distribution with DF = (1, n - 2).
The F-statistics and the p-value can be used to assess the significance of the regression models [3,4].

Akaike Information Criterion (AIC):

$$AIC = -2\ln(L) + 2(m+1)$$

where L is the maximum value of the likelihood for the model, and m is the number of parameters. For the simple regression modes, m will be 2, therefore,

$$AIC = -2\ln(L) + 2 \times 3$$

The best-fit model is the one that can explain the great amount of variation in response variable with the fewest explanatory variables. Thus, the accurate model has a smaller value of the AIC. The AIC allows us to determine the number of parameters in the model by comparing the AIC [5].
The variance inflation factor (VIF) for the jth explanatory variable:

$$VIF_j = \frac{1}{1 - {R_j}^2}$$

where R_j^2 is the R^2 on all the other explanatory variables. VIF can detect multicollinearity in the multiple regression models.
Multicollinearity is a phenomenon where one explanatory variable is highly correlated with one or more of the other explanatory variables in a multiple regression model, and it leads to undesirable consequences (e.g., coefficients may have unrealistic opposite sign, coefficients of slope are not stable; [4]).

3 References

1. Burnham KP, Anderson DR. Model Selection and Multimodel Inference: A Practical Information-Theoretic Approach. New York: Springer; 2002.
2. Bhattacharyya GK, Johnson RA. Statistical Concepts and Methods. Toronto, Canada: John Wiley and Sons; 1977.
3. Faraway JJ. Practical regression and ANOVA using R. Bath: University of Bath; 2002.
4. Helsel DR, Hirsch RM, Ryberg KR, Archfield SA, Gilroy EJ. Statistical methods in water resources. Virginia. U.S.: U.S. Geological Survey; 2020.
5. Diez DM, Barr CD, Cetinkaya-Rundel M. OpenIntro statistics Fourth Edition. OpenIntro; 2019.
